# Mutant prion proteins increase calcium permeability of AMPA receptors, exacerbating excitotoxicity

**DOI:** 10.1371/journal.ppat.1008654

**Published:** 2020-07-16

**Authors:** Elsa Ghirardini, Elena Restelli, Raffaella Morini, Ilaria Bertani, Davide Ortolan, Fabio Perrucci, Davide Pozzi, Michela Matteoli, Roberto Chiesa

**Affiliations:** 1 Laboratory of Pharmacology and Brain Pathology, Humanitas Clinical and Research Center, Rozzano—Milan, Italy; 2 Department of Neuroscience, Istituto di Ricerche Farmacologiche Mario Negri IRCCS, Milan, Italy; 3 Consiglio Nazionale delle Ricerche Institute of Neuroscience, Milan, Italy; University of Edinburgh, UNITED KINGDOM

## Abstract

Prion protein (PrP) mutations are linked to genetic prion diseases, a class of phenotypically heterogeneous neurodegenerative disorders with invariably fatal outcome. How mutant PrP triggers neurodegeneration is not known. Synaptic dysfunction precedes neuronal loss but it is not clear whether, and through which mechanisms, disruption of synaptic activity ultimately leads to neuronal death. Here we show that mutant PrP impairs the secretory trafficking of AMPA receptors (AMPARs). Specifically, intracellular retention of the GluA2 subunit results in synaptic exposure of GluA2-lacking, calcium-permeable AMPARs, leading to increased calcium permeability and enhanced sensitivity to excitotoxic cell death. Mutant PrPs linked to different genetic prion diseases affect AMPAR trafficking and function in different ways. Our findings identify AMPARs as pathogenic targets in genetic prion diseases, and support the involvement of excitotoxicity in neurodegeneration. They also suggest a mechanistic explanation for how different mutant PrPs may cause distinct disease phenotypes.

## Author summary

Genetic prion diseases are degenerative brain disorders caused by mutations in the gene encoding the prion protein (PrP). Different PrP mutations cause different diseases, including Creutzfeldt-Jakob disease, fatal familial insomnia and Gerstmann-Sträussler-Scheinker syndrome. How mutant PrP causes neuronal death and how different mutants encode distinct disease phenotypes is not known. Here we show that mutant PrP alters the subunit composition of glutamate AMPA receptors, promoting cell surface exposure of GluA2-lacking, calcium-permeable receptors, ultimately increasing neuronal vulnerability to excitotoxic cell death. We also demonstrate that the underlying molecular mechanism is the formation of a GluA2 subunit-PrP complex which is retained in the neuronal secretory pathway. PrP mutants associated with clinically different genetic prion diseases have distinct effects on GluA2 trafficking, depending on their tendency to misfold and aggregate in different intracellular organelles, indicating a possible contribution of this mechanism to the disease phenotype.

## Introduction

Synaptic dysfunction is an early process in prion disease, preceding synapse loss and neuronal death. Understanding the mechanisms of primary changes in synaptic function that lead to irreversible neurodegeneration has important implications for therapy. We describe morphological and functional alterations in neurons expressing prion protein (PrP) mutations associated with genetic prion disease, indicating a neurotoxic mechanism involving α-amino-3-hydroxy-5-methyl-4-isoxazolepropionic acid (AMPA) receptors (AMPARs).

Genetic prion diseases are rare and currently untreatable neurodegenerative disorders linked to mutations in the *PRNP* gene, encoding PrP, on chromosome 20 [1]. Approximately 70 pathogenic *PRNP* variants have been reported (see http://www.cureffi.org/2015/01/13/list-of-reportedly-pathogenic-prnp-variants/ for an up-to-date list), including missense mutations, expansions or deletions of a repeated sequence encoding an octapeptide motif in the N-terminal region, and stop codon mutations resulting in premature protein truncations.

One striking feature of genetic prion diseases is their phenotypic heterogeneity. Different *PRNP* mutations are associated with distinct disease subtypes, including genetic Creutzfeldt-Jakob disease (gCJD), fatal familial insomnia (FFI) and Gerstmann-Sträussler-Scheinker (GSS) syndrome [1]. The disease presentation can be influenced by *PRNP* polymorphism at codon 129, where either methionine (M) or valine (V) may be present. A noteworthy example is prion disease linked to the substitution of asparagine (N) for aspartic acid (D) at codon 178 which, depending on the amino acid at codon 129 on the mutant allele, segregates with either FFI (D178N/M129), primarily characterized by severe sleep disorders and autonomic dysfunction, or CJD^178^ (D178N/V129), clinically identified by global cortical dementia and motor abnormalities [2].

How mutant PrP causes neuronal death and how sequence variants of *PRNP* encode the information to specify distinct disease phenotypes is a central question in prion biology. PrP is a cell membrane glycoprotein highly expressed by neurons in the CNS. PrP is located at both pre- and post-synaptic sites, and there is ample evidence indicating a modulatory role in synaptic transmission which may be negatively affected by pathogenic mutations [3,4]. We previously found that PrP interacts physically with the α_2_δ-1 subunit of voltage-gated calcium channels (VGCCs) which govern depolarization-induced neurotransmitter release [5]. Mutant PrP misfolding and intracellular retention of α_2_δ-1 impaired synaptic delivery of VGCCs, and glutamatergic neurotransmission is disrupted in transgenic (Tg) mouse models of GSS and CJD^178^ [5].

PrP engages functional interactions with glutamate receptors, including AMPARs [6,7]. AMPARs are tetrameric, cation-permeable ionotropic receptors, which mediate the largest part of fast excitatory neurotransmission in the brain. Upon binding of glutamate, the pore opening allows the influx of Na^+^ ions and the efflux of K^+^ ions to depolarize the postsynaptic compartment. Depending on the subunit composition, AMPARs also allow influx of Ca^2+^. In the adult brain, the majority of GluA2-containing AMPARs are largely Ca^2+^-impermeable, due to a RNA editing that replaces a glutamine with a positively charged arginine in the pore-forming region of the assembled channel, thus preventing Ca^2+^ influx [8]. In contrast, GluA2-lacking AMPARs are Ca^2+^-permeable, and have higher single-channel conductance [9]. The Ca^2+^ permeability of AMPARs is thought to have important consequences for plasticity as well as cell viability (reviewed in [10]).

We explored the contribution of AMPAR dysfunction in genetic prion diseases by morphological and functional analyses in primary neurons from Tg mice expressing mouse homologs of the CJD^178^ and FFI mutations (moPrP D177N/V128 and moPrP D177N/M128), and of a nine-octapeptide repeat insertion (moPrP PG14) associated with GSS [11–14]. Membrane delivery of the GluA2 subunit of AMPARs was impaired in a PrP mutation-specific manner, with alterations in the structure, function and plasticity of the excitatory synapses. In addition, intracellular retention of GluA2 modified the subunit composition of AMPARs in the mutant neurons, increasing the number of GluA2-lacking, calcium-permeable AMPARs and resulting in greater calcium permeability and more vulnerability to excitotoxic cell death. These results cast fresh light on the mechanisms of neurodegeneration and phenotypic variability in genetic prion diseases.

## Results

### Synaptic structure and function are altered in hippocampal neurons expressing the FFI and CJD^178^ mutations

**Fig 1:**
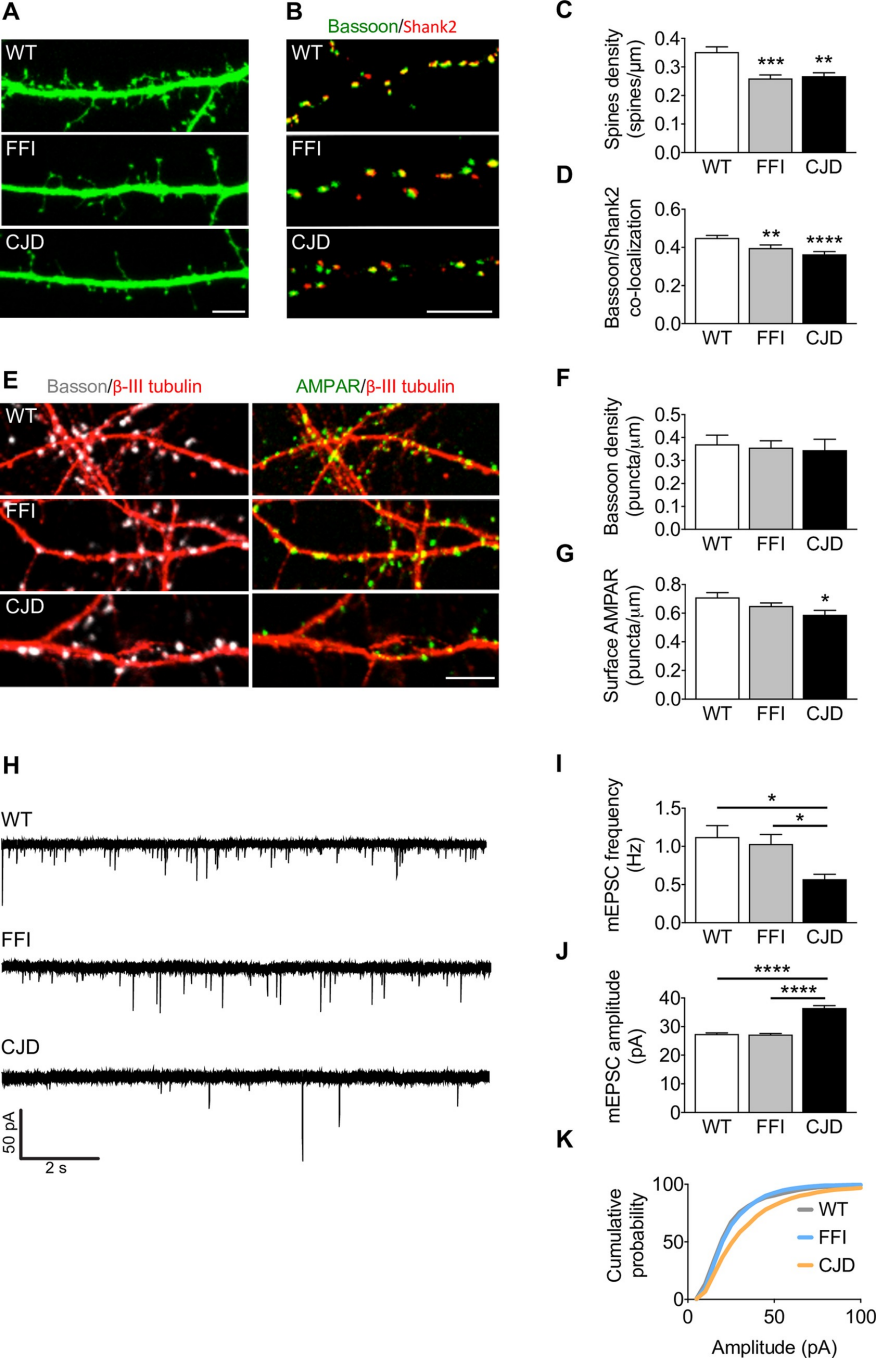
Synaptic structure and activity are altered in FFI and CJD hippocampal neurons. (A) Confocal representative images of WT, CJD and FFI neurons transfected with EGFP and (C) quantification of total spine density; 46–62 dendrites from 25–30 neurons for each condition. Kruskal-Wallis test followed by Dunn’s multiple comparison test: **p < 0.01, ***p < 0.001. (B) WT and mutant PrP neurons stained for Bassoon (green) and Shank2 (red) and (D) quantification of the levels of co-localization between the two; 69–87 fields for each condition, Kruskal-Wallis test followed by Dunn’s multiple comparison test: **p < 0.01, ****p < 0.0001. (E) WT, CJD and FFI neurons stained for Bassoon (gray), surface AMPAR (green) and β-III tubulin (red). Graphs show the number of Bassoon (F) or AMPAR (G) puncta per μm of dendrite; 17–23 dendrites from 10–15 neurons for each condition. One-way ANOVA followed by Tukey’s multiple comparison test: *p < 0.05. (H) Representative traces and analysis of mEPSC frequency (I) and cumulative amplitude (J, K). WT 32, FFI 25, CJD 18 cells. Kruskal-Wallis test followed by Dunn’s multiple comparison test: *p < 0.05, ****p < 0.0001. Data are the mean ± SEM of at least three independent experiments. Scale bars 5 μm in all images.

Dendritic spine loss has been described in prion-infected mice and organotypic cerebellar cultures, and in cultured hippocampal neurons exposed to PrP^Sc^, the infectious isoform of PrP [15–18]. We investigated whether synaptic alterations were also detectable in neurons expressing FFI or CJD^178^ PrP. We analyzed the morphology of excitatory synapses in primary cultures of hippocampal neurons from wild-type (WT), FFI and CJD mice at 13–15 days *in vitro* (DIV) after transfection with a plasmid encoding EGFP to allow visualization of dendritic spines. Both FFI and CJD neurons had less spine density than WT cells (Fig 1A and 1C). Consistent with this, the co-localization between the pre- and post-synaptic markers Bassoon and Shank2 was significantly lower in the mutant neurons (Fig 1B and 1D). The number of Bassoon puncta per μm was not altered in the mutant neurons, indicating that the defect involves mainly the post-synaptic compartment (Fig 1E and 1F).

To assess whether these structural alterations had functional consequences, we measured AMPAR-mediated activity by patch-clamp recordings. Despite the change in spine density (Fig 1A and 1C), FFI neurons displayed miniature excitatory postsynaptic currents (mEPSCs) comparable to those of WT cells (Fig 1H–1K) and similar density of surface AMPARs (Fig 1E and 1G). Conversely, CJD neurons had reduced amounts of plasma membrane AMPARs (Fig 1E and 1G) and a significantly lower frequency of mEPSCs (Fig 1H and 1I) with higher amplitude than controls (Fig 1H, 1J and 1K).

### Mutant PrP impairs membrane delivery of the GluA2 subunit of AMPA receptors

**Fig 2:**
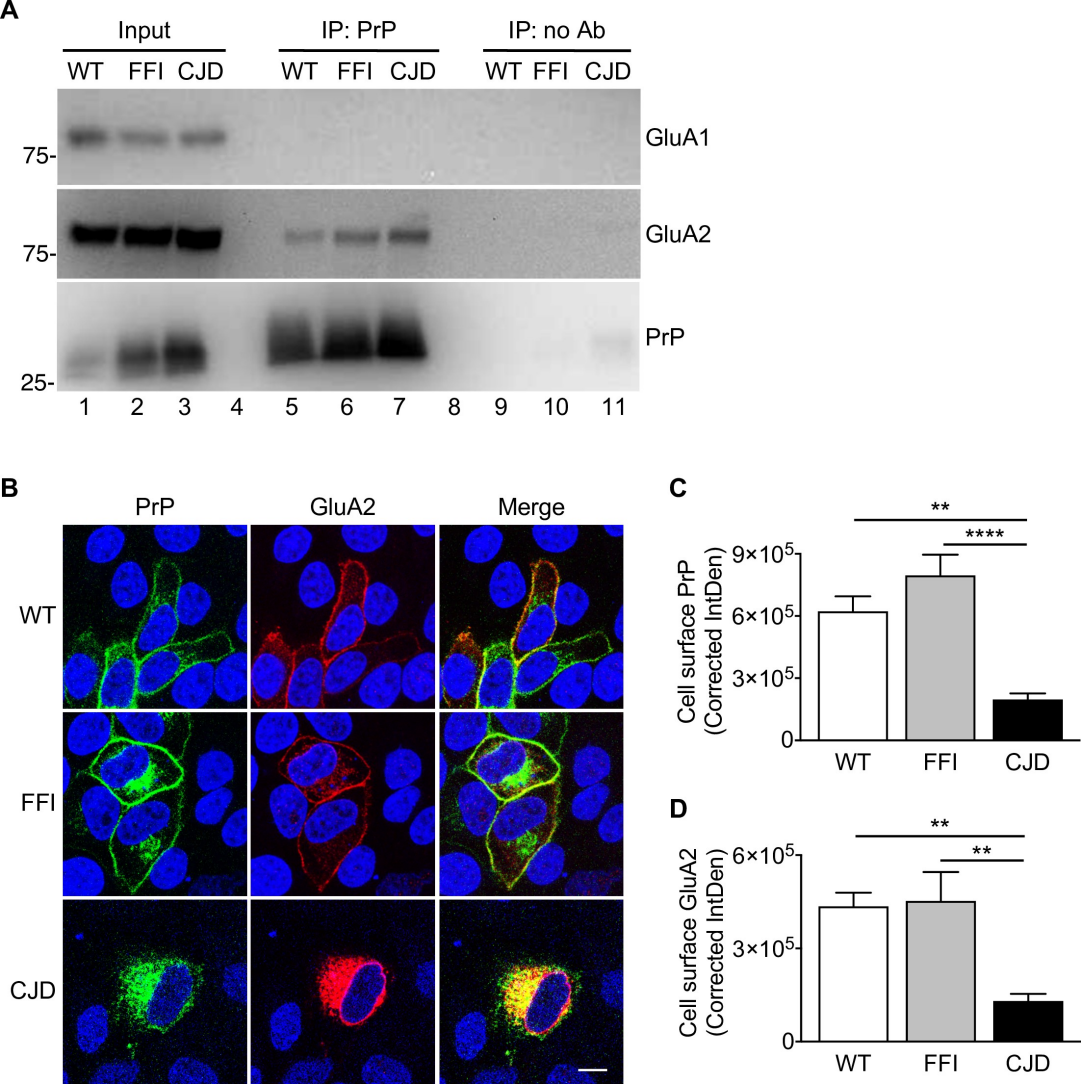
Mutant PrP interacts with the GluA2 AMPAR subunit impairing its membrane delivery. (A) Hippocampal protein extracts (500 μg) from WT, FFI and CJD mice were incubated with uncoated magnetic beads (IP: no Ab) or magnetic beads coated with anti-PrP monoclonal antibody 94B4 (IP: PrP). The immunoprecipitated proteins were analyzed by western blot with anti-GluA1 or anti-GluA2 antibody or anti-PrP polyclonal antibody P45-66. The input is 20 μg of total proteins. Lanes 4 and 8 were left empty on purpose. This experiment is representative of three similar ones. (B) HeLa cells were co-transfected with plasmids encoding WT, FFI or CJD PrP-EGFP fusion protein, and the AMPAR subunit GluA2. After 48h, cells were fixed, permeabilized, stained with anti-GluA2 (red) antibody, and reacted with Hoechst 33258 (blue) to stain the nuclei. Scale bar 10 μm. (C, D) The fluorescent density of PrP and GluA2 on the cell surface was measured and background corrected (Corrected IntDen). Each bar indicates the mean ± SEM of 12–17 tranfected cells from three independent experiments. **p < 0.01, ****p < 0.0001 by one-way ANOVA, Tukey’s post hoc test.

PrP interacts physically with AMPARs [6,7]. To assess whether pathogenic PrP mutations influenced this interaction, we immunoprecipitated PrP from hippocampal homogenates of WT, FFI and CJD mice and immunoblotted the precipitated proteins with antibodies against GluA1 and GluA2, the two main AMPAR subunits expressed at hippocampal synapses. The WT and mutant PrPs co-immunoprecipitated with GluA2 but not with GluA1 (Fig 2A).

CJD and FFI PrPs misfold and aggregate in the secretory pathway and are partly retained in the endoplasmic reticulum (ER) and Golgi [11,14,19–21]. To see whether GluA2 was retained intracellularly with these mutant PrPs, we co-transfected HeLa cells with plasmids encoding GluA2 and EGFP-tagged WT or mutant PrP. After 48h, cells were immunostained with antibodies against GluA2, and analyzed by fluorescence confocal microscopy. The majority of WT PrP localized on the cell surface, while large fractions of the mutant PrPs were found in intracellular compartments (Fig 2B and 2C). In line with previous results [11,14,21,22], FFI PrP showed an intracellular distribution consistent with accumulation in the Golgi and was more efficiently expressed on the cell surface than CJD PrP, which showed a perinuclear distribution indicative of ER retention (Fig 2B and 2C). In cells expressing WT PrP, GluA2 was efficiently expressed on the plasma membrane (Fig 2B and 2D) where the two proteins co-localized largely (S1 Fig), consistent with their association in cholesterol-rich microdomains [23–27]. Quantitative analysis of plasma membrane GluA2 fluorescence indicated that cell surface expression of GluA2 was similar in WT and FFI but reduced ~70% in CJD PrP-expressing cells (Fig 2B and 2D).

### AMPA receptor trafficking is impaired in CJD but not FFI hippocampal neurons

**Fig 3:**
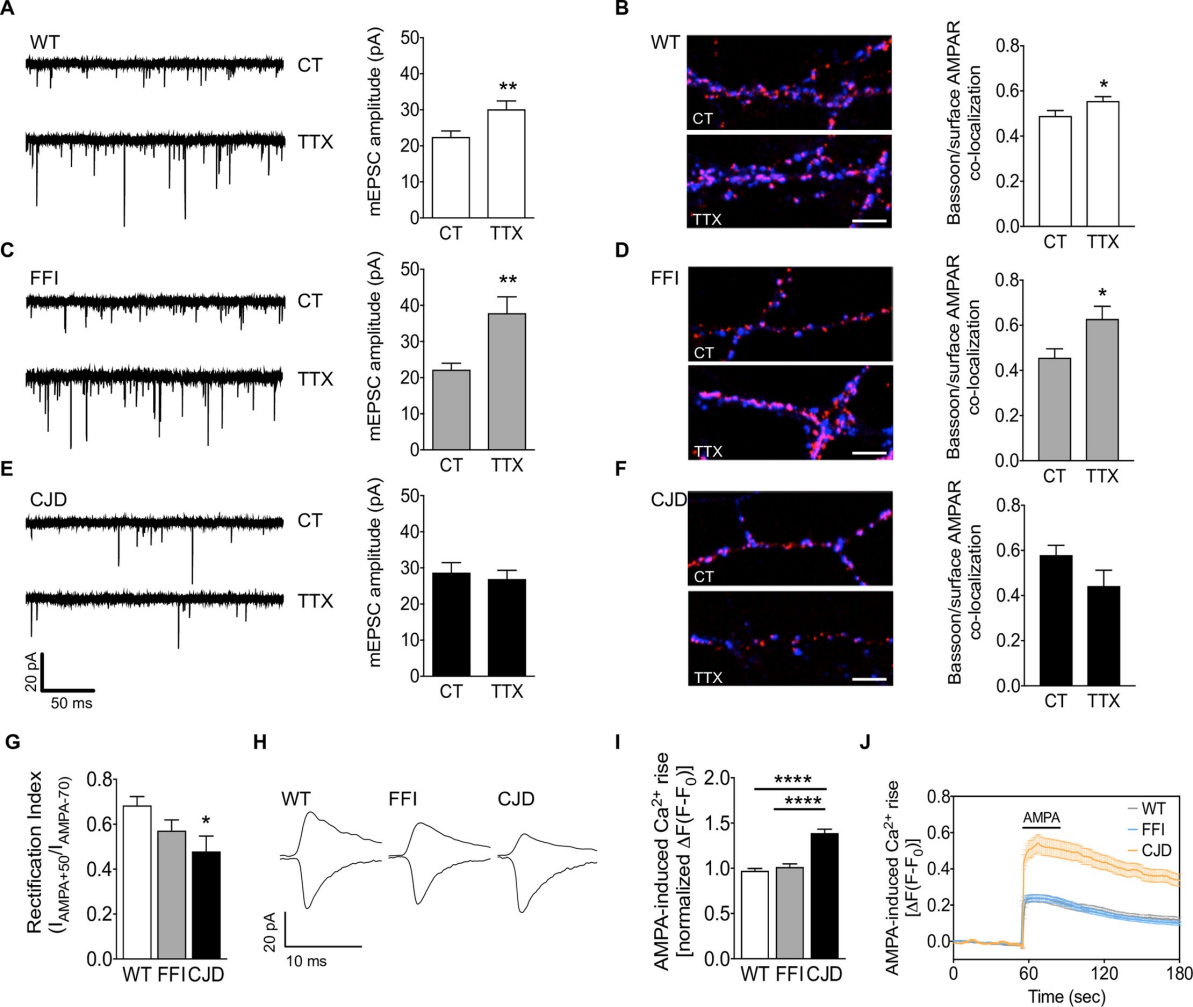
AMPA receptor trafficking is impaired in CJD but not in FFI neurons. (A, C, E) Representative traces and quantification of mEPSC amplitude in control and TTX-treated WT, FFI and CJD neurons. WT 16 (CT) and 18 (TTX), FFI 12 (CT) and 13 (TTX), CJD 18 (CT) and 16 (TTX) cells. Mann-Whitney test: **p < 0.01. (B, D, F) Confocal representative images and quantification of the levels of co-localization between Bassoon (blue) and surface AMPAR (red) in the conditions described above. Scale bar 5 μm. Unpaired Student’s t-test: *p < 0.05. (G) Rectification index (I_AMPA+50_/I_AMPA-70_) and (H) representative traces in WT and mutant neurons. WT 24, FFI 15, CJD 7 cells. Kruskal-Wallis test followed by Dunn’s multiple comparison test: *p < 0.05. (I) Analysis of calcium peaks and (J) representative traces of calcium response to AMPA (30 μM) in WT, FFI and CJD neurons. Six fields (79–150 responding cells) for each condition. Kruskal-Wallis test followed by Dunn’s multiple comparison test: ****p < 0.0001. Data are the mean ± SEM of at least three independent experiments.

To assess AMPAR trafficking in mutant PrP neurons we used a well-established homeostatic plasticity protocol, which induces selective targeting of AMPARs to the synapses [28]. Primary hippocampal neurons were treated with tetrodotoxin (TTX) for 48h and the function and distribution of synaptic AMPARs was assessed by electrophysiology and confocal microscopy. In line with previous reports [28–30], the chronic blockade of neuronal firing significantly increased mEPSC amplitude in WT neurons (Fig 3A), accompanied by increased co-localization between the pre-synaptic marker Bassoon and surface AMPARs (Fig 3B), indicative of post-synaptic potentiation. Results were similar for FFI neurons, in which TTX significantly increased both mEPSC amplitude and Bassoon/AMPAR co-localization (Fig 3C and 3D). In contrast, this protocol did not alter mEPSC amplitude or the co-localization of pre- and post-synaptic markers in CJD neurons (Fig 3E and 3F), suggesting defective insertion of AMPARs into the synaptic membrane.

Since GluA2 interacts with PrP and is almost completely retained intracellularly in CJD PrP-expressing cells (Fig 2A and 2B), we employed the rectification properties of GluA2 to analyze the presence of this subunit in synaptic AMPARs. GluA2-lacking receptors are strongly rectifying whereas GluA2-containing receptors are not [29,31], so we measured the rectification index (RI) of AMPARs by recording mEPSCs at -70 and +50 mV and calculating the ratio of current amplitude. The RI in CJD neurons was significantly smaller than in WT and FFI cells, indicating a larger synaptic population of GluA2-lacking AMPARs (Fig 3G and 3H).

Since GluA2 renders AMPARs impermeable to calcium, we reasoned that a decrease in expression of this subunit could result in increased calcium influx upon AMPAR activation. To verify this, we measured the intracellular calcium transients induced by 30 μM AMPA. The AMPA-induced calcium elevation was significantly larger in CJD than WT and FFI neurons (Fig 3I and 3J). Consistent with this evidence, the selective inhibitor of GluA2-lacking AMPARs IEM-1460 significantly reduced AMPA-induced neuronal death in CJD but not FFI neurons (S2 Fig). These data indicate that CJD PrP affects AMPAR subunit composition, resulting in a larger proportion of GluA2-lacking, calcium-permeable AMPARs.

### Cerebellar granule neurons expressing PG14 PrP are hypersensitive to AMPAR-mediated excitotoxicity

In Tg(PG14) mice abnormalities in cerebellar neurotransmission due to mutant PrP accumulation in the ER precede massive apoptotic degeneration of cerebellar granule neurons (CGNs) [5,13]. We therefore reasoned that this model could be the most suitable to test whether functional alterations in AMPARs due to intracellular retention of GluA2 contribute to mutant PrP-induced neuronal cell death.

**Fig 4:**
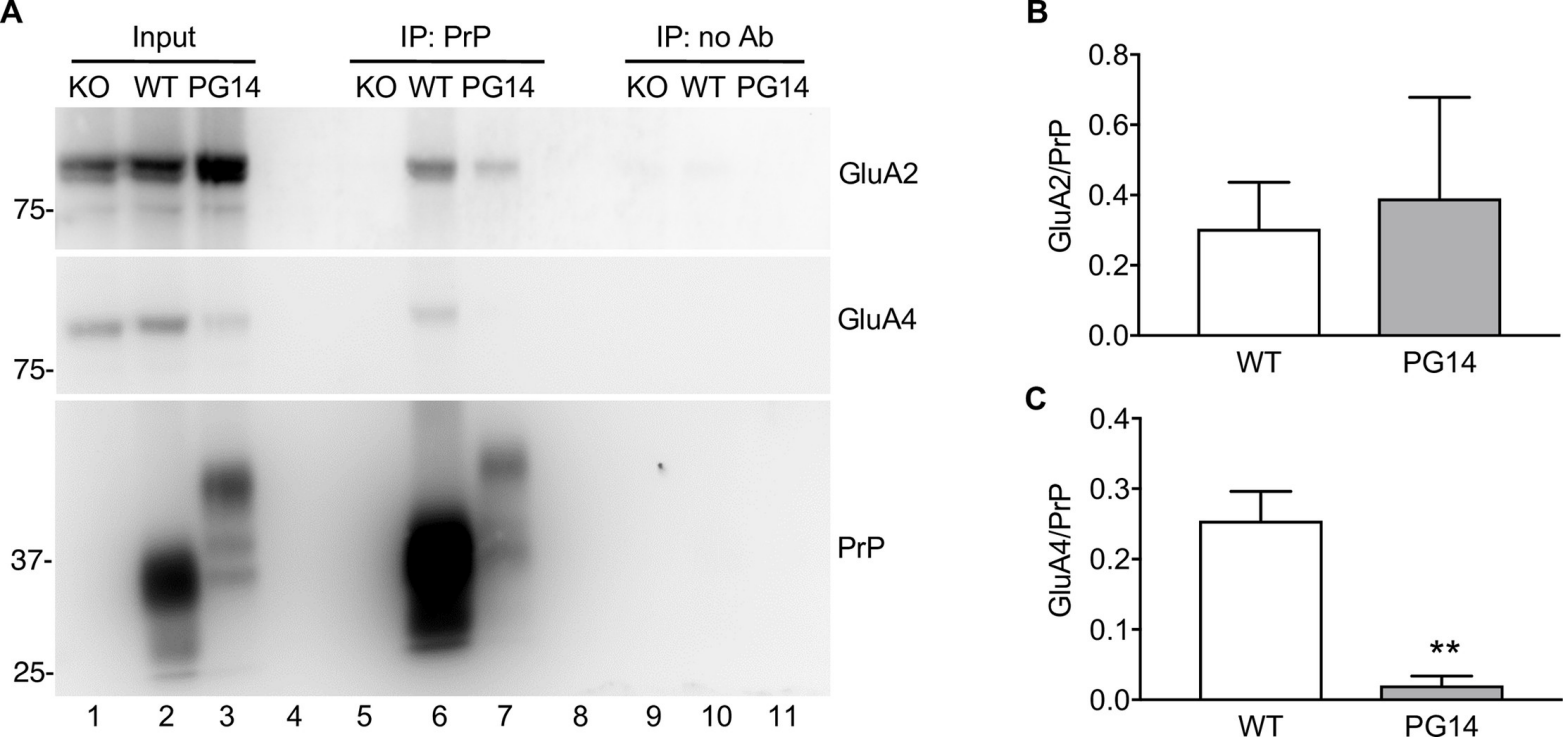
PrP and GluA2 co-immunoprecipitate from cerebellar extracts. (A) Cerebellar protein extracts (500 μg) from Tg(WT), Tg(PG14) and PrP knockout (KO) mice were incubated with uncoated magnetic beads (IP: no Ab) or magnetic beads coated with anti-PrP monoclonal antibody 94B4 (IP: PrP). The immunoprecipitated proteins were analyzed by western blot with anti-GluA2 or anti-GluA4 antibody or anti-PrP polyclonal antibody P45-66. The input is 20 μg of total proteins. (B, C) The amounts of immunoprecipitated GluA2 (B) and GluA4 (C) were quantified by densitometry of western blots like in (A) and normalized on the amount of immunoprecipitated PrP. Data are the mean ± SEM of 3–4 replicates from three independent experiments; **p < 0.01 by Student’s t-test.

First we tested whether PG14 PrP interacts with AMPARs. We immunoprecipitated PrP from cerebellar extracts of WT and PG14 mice, and immunoblotted the precipitated fractions with antibodies against GluA2 and GluA4, which are the AMPAR subunits specifically expressed in CGNs. Both WT and PG14 PrP co-immunoprecipitated with GluA2 (Fig 4A and 4B). WT PrP co-immunoprecipitated also with GluA4, whereas PG14 PrP interacted much less efficiently with this subunit (Fig 4A and 4C).

**Fig 5:**
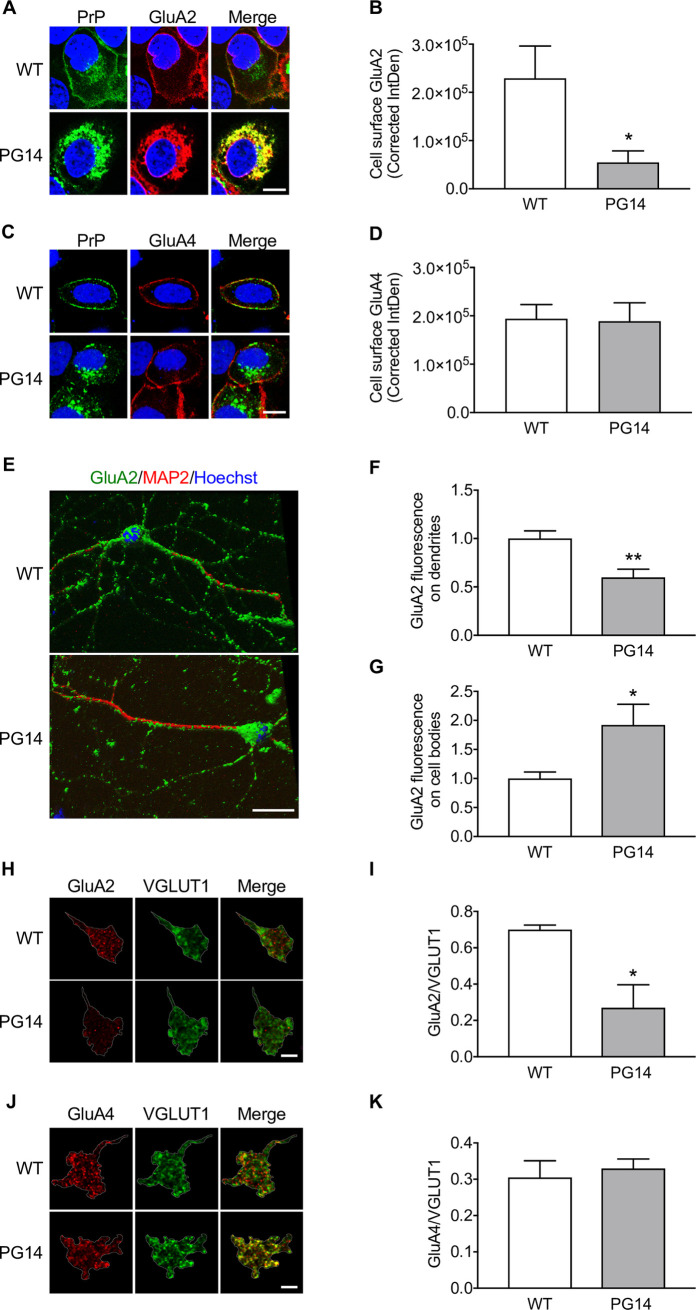
GluA2 is retained intracellularly and is expressed less at post-synaptic sites in the granule cell layer of Tg(PG14) mice. (A) HeLa cells were co-transfected with plasmids encoding WT or PG14 PrP-EGFP fusion protein, and the AMPAR subunit GluA2. After 48h cells were fixed, permeabilized, stained with anti-GluA2 (red), and reacted with Hoechst 33258 (blue) to stain the nuclei. (B) The fluorescent density of GluA2 on the cell surface was measured and corrected for background (Corrected IntDen). Each bar indicates the mean ± SEM of 12 WT and 14 PG14 PrP-transfected cells from three independent experiments; *p<0.01 by Student’s t-test. (C) HeLa cells were co-transfected with WT or PG14 PrP and EGFP-tagged GluA4. After 48 h cells were fixed, permeabilized, stained with anti-PrP monoclonal antibody 98A3 (green), and reacted with Hoechst 33258 (blue) to stain the nuclei. (D) The fluorescent density of GluA4 on the cell surface was measured and background corrected. Each bar indicates the mean ± SEM of 6 WT and 6 PG14 PrP-transfected cells. (E) CGNs from WT and PG14 mice were fixed, permeabilized and immunostained with mouse monoclonal anti-GluA2 (green) and chicken polyclonal anti-MAP2 (red). Cells were reacted with Hoechst 33258 (blue) to stain the nuclei, and analyzed by confocal microscopy using sequential Z-stack acquisition mode and 3D reconstruction. The GluA2 signal on dendrites (F) and cell bodies (G) was analyzed by NIH ImageJ software. Data are the mean ± SEM of 18 WT cells (28 dendrites) and 18 PG14 cells (27 dendrites) from three independent experiments. (H-K) Brain sections of WT and PG14 mice were stained with anti-GluA2 (H) or GluA4 (J) (red) and anti-VGLUT1 (green) antibodies. The GluA2 (I) and GluA4 (K) in each cerebellar glomerulus were measured and expressed as the ratio between the area of the AMPA receptor subunit and VGLUT1 signals. The dotted line identifies a single glomerulus used for quantification. Signal outside the ROI has been cleared with ImageJ for clarity. Bars indicate the mean ± SEM of three mice per group (1098–1392 glomeruli). *p < 0.05 by Student’s t-test. Scale bars 20 μm in A, C and E; and 125 μm in H and J.

Next, we tested whether the distribution of AMPARs was altered in cells expressing PG14 PrP. HeLa cells were co-transfected with plasmids encoding GluA2 and GluA4, and either WT or PG14 PrP (untagged or EGFP-tagged). After 48h cells were immunostained with antibodies against the AMPAR subunits (or against PrP when using EGFP-tagged AMPARs and untagged PrPs) and analyzed by fluorescence confocal microscopy. Consistent with previous results [5], the majority of WT PrP localized on the cell surface, whereas PG14 PrP was mostly found in intracellular compartments (Fig 5A and 5C). In cells expressing WT PrP, GluA2 was efficiently expressed on the plasma membrane (Fig 5A and 5B). In contrast, it was weakly expressed on the surface of PG14 PrP-transfected cells and was mostly found in PrP-positive perinuclear patches (Fig 5A and 5B). Consistent with its weak interaction with PG14 PrP (Fig 4), GluA4 was not retained intracellularly, and was efficiently delivered to the plasma membrane like in WT PrP-expressing cells (Fig 5C and 5D).

GluA2 distribution was also altered in primary CGNs from PG14 mice, where immunoelectron microscopy confirmed ER retention of mutant PrP and showed swelling of the ER cisternae, consistent with protein accumulation in this transport organelle (S3 Fig). Quantitative immunofluorescence analysis showed that GluA2 was significantly less present on the neurites and more concentrated in the soma of PG14 neurons compared to WT cells (Fig 5E–5G). Immunofluorescence analysis of the cerebellar granule cell layer of PG14 mice found reduced amounts of GluA2 in glomeruli, the VGLUT1-immunopositive structures characteristic of glutamatergic mossy fiber synapses onto post-synaptic granule cell dendrites [32], but no change in GluA4 (Fig 5H–5K).

**Fig 6:**
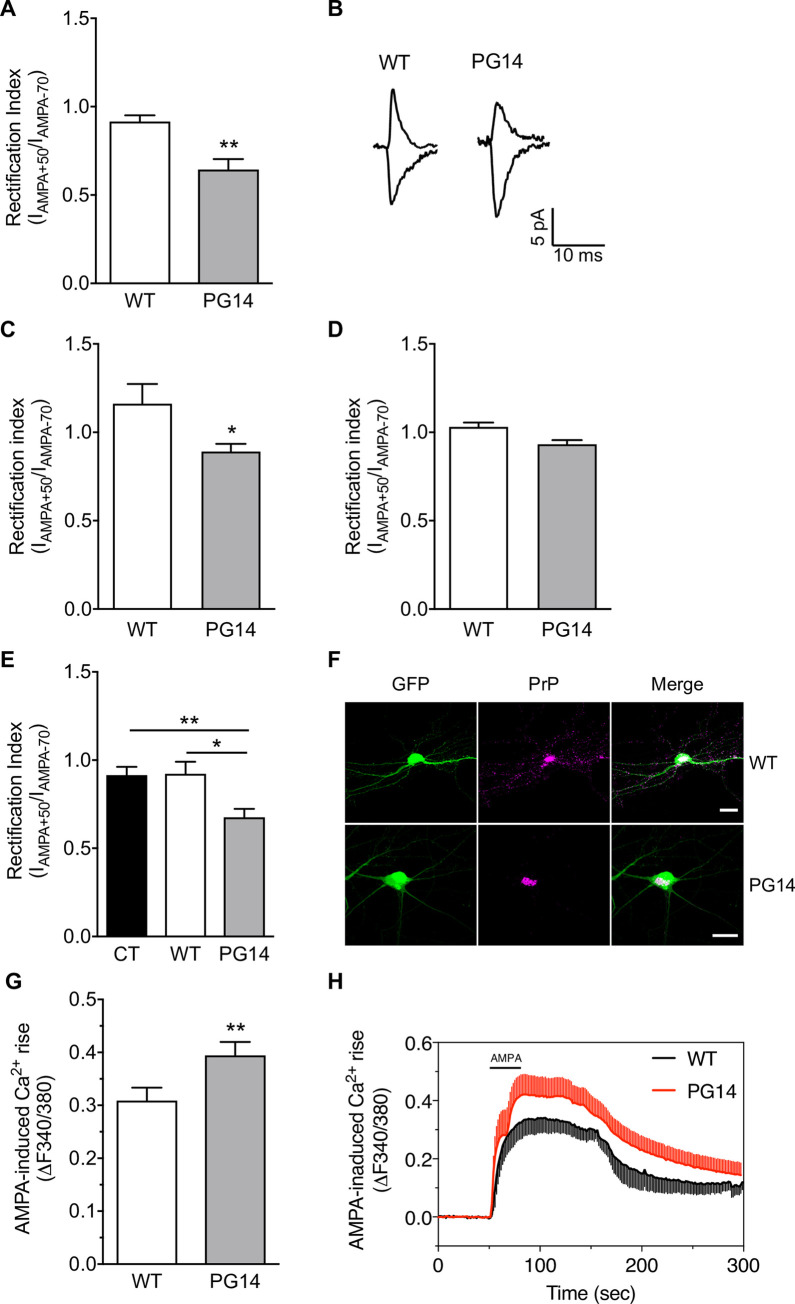
PG14 PrP alters AMPA receptor subunit composition and calcium permeability in primary CGNs and transfected hippocampal cells. (A) Rectification index (I_AMPA+50_/I_AMPA-70_) and (B) representative traces obtained by whole-cell patch-clamp experiments in WT and PG14 cultured cerebellar granule cells. I_-70mV_/I_+50mV_: WT 18, PG14 21. **p<0.01 by Mann-Whitney test. Rectification index recorded from cerebellar granule (C) or Purkinje (D) cells in acute brain slices. I_-70mV_/I_+50mV_ granule cells: WT 8 cells from 3 mice; PG14 11 cells from 4 mice. I_-70mV_/I_+50mV_ Purkinje cells: WT 4 cells from 3 mice; PG14 4 cells from 3 mice. * p<0.05 by Unpaired T-test. (E) Rectification index (I_AMPA+50_/I_AMPA-70_) in hippocampal neurons transfected with the pBud-CE4.1 vector expressing only GFP (CT), or also expressing WT or PG14 PrP. CT 18, WT 9, PG14 16 cells. Kruskal-Wallis test followed by Dunn’s multiple comparison test: *p < 0.05; **p < 0.01. (F) Confocal images showing primary hippocampal neurons from C57BL/6J mice transfected with a bigenic plasmid that drives efficient PrP and GFP expression. Scale bar 10 μm. (G) Analysis of calcium peaks and (H) representative traces of calcium response to AMPA (30 μM) in WT and PG14 cerebellar granule neurons. Six fields (102–123 responding cells) for each condition. **p < 0.01 by Mann-Whitney test. Bar graphs show mean ± SEM of at least of three independent experiments.

Like in CJD neurons (Fig 3G and 3H), in cultured PG14 CGNs too the RI was significantly smaller than in WT controls (Fig 6A and 6B), indicating reduced GluA2-containing AMPARs at PG14 synapses. Electrophysiological analysis in acute brain slices confirmed the reduced RI in PG14 cerebellar granules (Fig 6C), but found no alterations in Purkinje cells (Fig 6D), consistent with the lack of mutant PrP expression in the latter [12,33].

To demonstrate that expression of PG14 PrP was directly responsible for GluA2 mislocalization, we transfected primary hippocampal neurons from C57BL/6J mice with a bigenic plasmid that drives efficient PrP and GFP expression [5], and measured current amplitudes at +50 and -70 mV in GFP-positive cells (Fig 6E and 6F). PG14 PrP-transfected neurons had a significantly smaller RI than empty plasmid- or WT PrP-transfected cells (Fig 6E), indicating that acute PG14 PrP expression was sufficient to alter AMPAR composition.

Next we measured calcium influx in response to AMPA. In the presence of AP5, cadmium and nifedipine to block N-methyl-D-aspartate (NMDA) receptors, high-voltage activated and L-type calcium channels, IEM-1460 reduced AMPA-induced calcium rise in WT CGNs, indicating that these cells express basal levels of GluA2-lacking AMPARs (S4 Fig). Consistent with reduced GluA2-contaning AMPARs in PG14 cells, calcium influx in response to AMPA was higher in PG14 than in WT CGNs (Fig 6G and 6H). Thus, like in CJD neurons, GluA2 accumulates intracellularly in PG14 neurons, resulting in post-synaptic expression of GluA2-lacking, calcium-permeable AMPARs.

**Fig 7:**
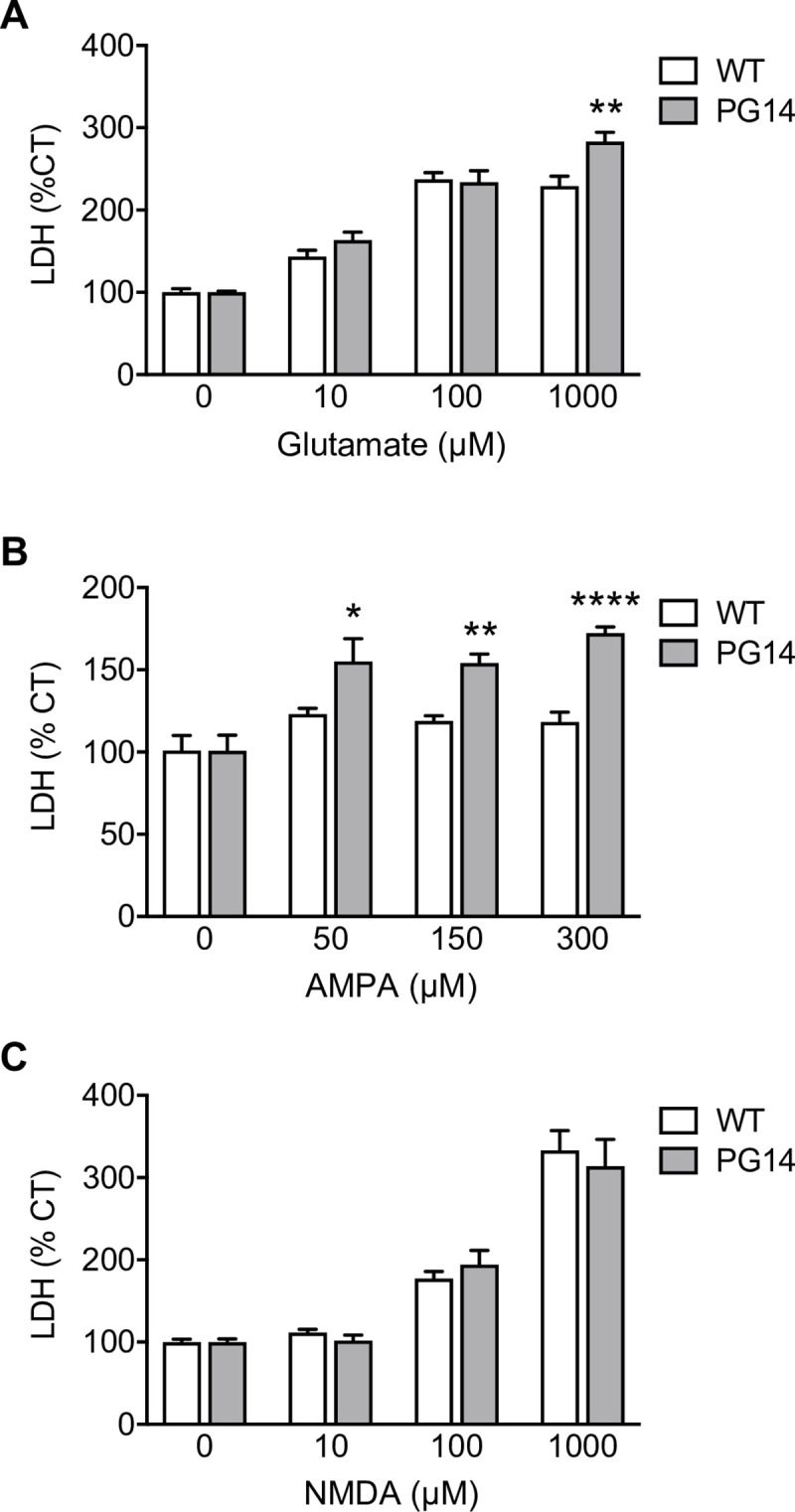
PG14 CGNs are hypersensitive to glutamate- and AMPA- but not NMDA-induced excitotoxicity. CGNs from WT and PG14 mice were treated with glutamate (A), AMPA (B) or NMDA (C) at the concentrations indicated. After 24h cell death was quantified by LDH assay, and expressed as a percentage of the values for cells treated with the vehicle. Data are the mean ± SEM of 6–11 replicates from three independent experiments; *p < 0.05, **p < 0.01, ****p < 0.0001 by two-way ANOVA, Bonferroni’s post-hoc test.

To test whether the defective delivery of GluA2 subunits to the cell surface sensitized cells to glutamate-induced, calcium-mediated toxicity, we exposed primary CGN cultures from WT and PG14 mice to glutamate, AMPA or NMDA. After 24h we measured the activity of lactate dehydrogenase (LDH), which is released in the culture medium by dying cells. PG14 CGNs were significantly more vulnerable than WT cells to the toxicity of glutamate and AMPA, but not NMDA (Fig 7), consistent with AMPAR-mediated excitotoxic cell death. In line with evidence in cortical neurons [34], we observed increased numbers of TUNEL-positive cells in primary CGNs exposed to AMPA (S5 Fig), supporting the idea that AMPAR-mediated excitotoxicity may contribute to CGN apoptosis *in vivo*.

**Fig 8:**
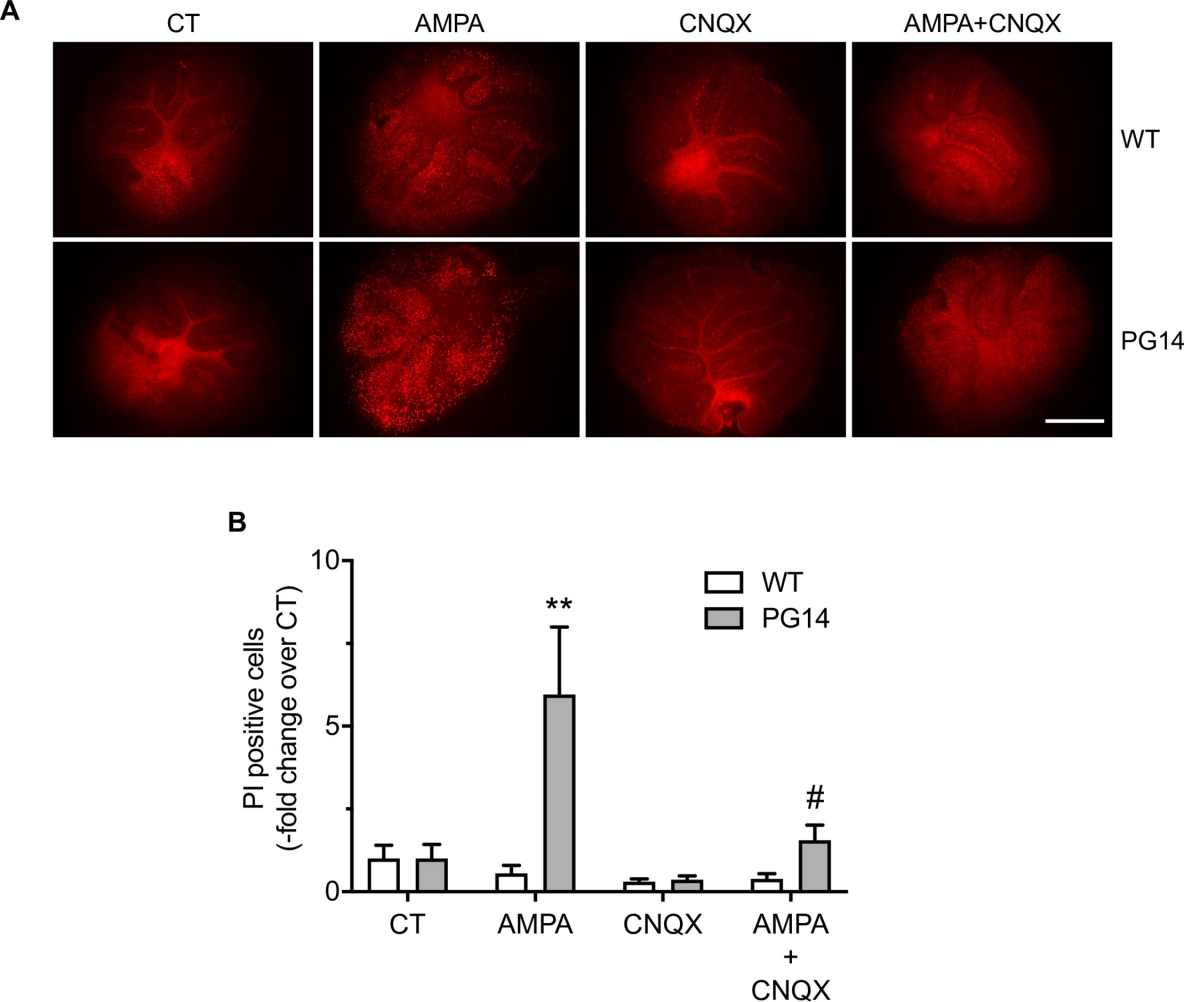
Cultured organotypic cerebellar slices from Tg(PG14) mice show increased vulnerability to AMPA toxicity. (A) Organotypic cerebellar slices from WT and PG14 mice were cultured for 15 days before exposure to 50 μM AMPA, 25 μM CNQX, or AMPA and CNQX, for 24h. Scale bar 500 μm. (B) The PI-positive cells were counted after 24h and expressed as the–fold change from vehicle-treated cells (CT). Data are the mean ± SEM of 9–24 cerebellar slices from three independent experiments. **p < 0.01 and *vs*. PG14 CT; ^#^p < 0.05 *vs*. PG14 AMPA by two-way ANOVA, Tukey’s post-hoc test.

To determine whether PG14 CGNs had greater susceptibility to excitotoxicity in a more physiological context, we used cerebellar organotypic slice cultures. Cerebellar slices were prepared from 9-day-old mice and cultured for 15 days before exposure to 50 μM AMPA with or without the competitive AMPAR antagonist 6-cyano-7-nitroquinoxaline-2,3-dione (CNQX). Propidium iodide (PI)-positive cells (i.e. dead) were counted after 24h. AMPA increased cell mortality 10-fold in PG14 but not in WT slices, and this was significantly reduced by co-treatment with CNQX (Fig 8).

## Discussion

The present study found that mutant PrP affects intracellular trafficking of GluA2, leading to cell surface exposure of GluA2-lacking, calcium-permeable AMPARs and increased vulnerability to excitotoxic, calcium-mediated cell death. We documented a physical interaction between PrP and AMPAR subunits and found that PrP mutants which tended to accumulate in different compartments of the secretory pathway had distinct effects on GluA2 trafficking and excitatory neurotransmission. These results, in conjunction with evidence of dendritic spine alterations in mutant PrP neurons, point to altered post-synaptic transmission and glutamate excitotoxicity in neurodegeneration, and suggest that mutation-specific alterations of AMPAR function may contribute to selective neuronal vulnerability in genetic prion disease.

### Intracellular retention of mutant PrP and alteration of AMPA receptor trafficking

PrP and GluA2 interacted physically by co-immunoprecipitation, confirming previous observations [6,7], and we document a new PrP interaction with GluA4 in the cerebellum. In contrast to Watt et al. [7], who found that WT PrP co-immunoprecipitates also with GluA1 and that the D177N mutation disrupts interaction with both GluA1 and GluA2, we did not see any co-immunoprecipitation with GluA1, or any effect of pathogenic mutations on PrP interaction with GluA2. The reason for this difference is not clear: it may be due to the use of co-immunoprecipitation procedures with different stringencies, and/or to the presence of zinc chelators in the co-immunoprecipitation buffer used by Watt et al., which may alter zinc-dependent tertiary contacts between the globular C-terminal domain and the flexible N terminus of PrP involved in binding to AMPARs [7,35].

We did find, however, that PG14 co-immunoprecipitates with GluA4 much less efficiently than WT PrP. Epitope scanning indicated that the N-terminal polybasic region required for interaction with AMPARs [7] is less exposed to the solvent in PG14 than in WT or D177N PrP [36]. Thus the nine-octapeptide repeat insertion may produce a specific structural change in the PrP N terminus which selectively disrupts interaction with GluA4.

GluA2, but not GluA4, was intracellularly retained in HeLa cells expressing PG14 PrP. Smaller amounts of GluA2 were detected on neurites of cultured CGNs and in cerebellar glomeruli of PG14 mice, whereas the synaptic content of GluA4 did not change. The fact that PG14 PrP interacts physically with GluA2 but not GluA4 suggests a mechanism by which interaction between mutant PrP and GluA2 results in the latter being sequestered in secretory organelles, impairing GluA2 assembly in the channel complex and its delivery to synaptic sites.

Although all the PrP mutants we analyzed interacted with GluA2, they had different effects on AMPAR trafficking. CJD and PG14 PrPs caused marked intracellular retention of GluA2 in HeLa cells, whereas FFI PrP had a subtler effect, with a much larger fraction of molecules reaching the plasma membrane. This held true in primary neurons, where immunofluorescence staining and analysis of the rectification index indicated reduced amounts of GluA2-containing AMPARs on neurites of CJD and PG14 but not FFI cells. We previously found that CJD and PG14 PrPs are preferentially retained in the ER, while FFI PrP accumulates mainly in the Golgi [11,14,21,22], perhaps because organelle-specific factors such as ER chaperones or differences in pH influence the intrinsic misfolding tendency of these mutants in different ways [36–39].

AMPA receptors assemble in the ER, where quality control mechanisms ensure correct subunit folding and assembly before export to post-ER compartments and delivery to the plasma membrane where a subpopulation resides in cholesterol-rich microdomains, like PrP [23–27,40–42]. It is possible that GluA2 and PrP interact transiently in the ER, and the CJD and PG14 mutants retain GluA2 subunits because their exit from this organelle is delayed [19], whereas FFI PrP, which transits the ER more efficiently, has a milder effect. Supporting this, in CJD and PG14 PrP-expressing HeLa cells GluA2 had a perinuclear/reticular distribution, indicative of ER retention, which largely overlapped with PrP, whereas in FFI PrP-expressing cells GluA2 did not co-localize with intracellular PrP and was efficiently delivered to the plasma membrane.

Different degrees of intracellular GluA2 retention had different functional consequences in mutant PrP neurons. This emerged clearly from electrophysiological analyses of primary hippocampal neurons from CJD and FFI mice. Only the former presented excitatory miniature events with lower frequency and larger amplitude, in line with reduced surface AMPAR density and increased single-channel conductance due to lack of GluA2 [43]. CJD but not FFI neurons were impaired in synaptic scaling in response to TTX, a homeostatic plasticity process that requires plasma membrane insertion of GluA2-containing AMPARs [29,44,45]. Finally, CJD neurons had a lower rectification index than WT and FFI cells, a finding directly attributable to the low synaptic content of GluA2 [29,31].

Consistent with expression of GluA2-lacking, calcium-permeable AMPARs, CJD and PG14 neurons had a bigger calcium rise in response to AMPA. Considering that excessive activity-dependent calcium influx may result in excitotoxicity [46–48], this suggested that increased AMPAR-mediated excitation might contribute to cell death. Our tests in primary neurons and organotypic cerebellar slices from CJD and PG14 mice supported this, showing enhanced vulnerability to glutamate and AMPA, and rescue by IEM-1460 or CNQX. Importantly, NMDA toxicity towards PG14 CGNs was not enhanced, indicating that they were not generally more sensitive to toxic insults. It will now be important to test whether AMPAR antagonists prevent neuronal death and relieve neurological illness in the mutant mice. Perampanel, a non-competitive AMPAR antagonist already used in humans to treat seizures, would be a good candidate to test.

AMPAR-mediated excitotoxicity may contribute to selective neurodegeneration in other inherited neurodegenerative disorders, such familial amyotrophic lateral sclerosis linked to *C9ORF72* repeat expansion, in which GluA1 expression increases specifically in motor neurons [49]. In some other genetic neurodegenerative conditions, such as Niemann-Pick disease type C1, an increase in calcium-impermeable AMPARs due to GluA2 up-regulation may instead play a role [50].

### Dendritic spine alterations in mutant PrP neurons

Both FFI and CJD neurons present structural alterations of the excitatory synapses, with lower dendritic spine density and less co-localization between pre- and post-synaptic markers. Spine pathology is an early manifestation of prion infection attributable to a toxic effect of PrP^Sc^ mediated by endogenous PrP but independent of prion replication [16–18,51]. Our data indicate that synaptic alterations also occur in models of genetic prion disease in which neuronal pathology is caused by abnormal forms of PrP structurally distinct from PrP^Sc^ [11,36,52,53]. These findings support the central role of synaptic pathology in prion disease which is most likely attributable to cell-autonomous mechanisms triggered by misfolding of endogenous PrP [3].

AMPA receptor trafficking has been proposed as one of the primary driving forces in spine morphogenesis [54], with the N-terminal domain of GluA2 a direct modulator of spine growth [55]. Intracellular retention of GluA2 may therefore explain, at least in part, the low spine numbers in mutant PrP neurons. PrP mediates Aβ oligomer-induced dendritic spine degeneration via a signaling cascade involving the metabotropic glutamate receptor mGluR5 and the kinase Fyn [56]. However, this signaling does not appear to contribute to PrP^Sc^-induced dendritic spine degeneration which, rather, requires AMPA and NMDA receptor activation and p38 mitogen-activated protein kinase (MAPK) signaling [17]. It will be interesting to see whether p38 MAPK is activated in neurons expressing mutant PrP and inhibitors of this signaling rescue the dendritic spine alterations, like in neurons exposed to PrP^Sc^ [17].

Surprisingly, the reduction in the number of dendritic spines and in co-localization between pre- and post-synaptic compartments does not appear to have any important effect on basal excitatory neurotransmission in FFI neurons. This suggests that structural synaptic alterations are not always paralleled by functional impairment, and further points to the specific defect of AMPARs as the main phenotypic driver. However, GluA2 retention is likely to be just one of the pathological events at mutant PrP synapses, and other mechanisms may be responsible for FFI neuropathology. PrP in fact has a wide interactome that includes several other synaptic proteins [57]. Intracellular accumulation of FFI PrP may lead to functional perturbation of synaptic proteins that are preferentially expressed in certain neurons of the brain. While our study employed hippocampal neurons as a well-established model of synaptic plasticity and function, FFI primarily affects neurons in the thalamus and inferior olives, and it is possible that FFI-specific phenotypes may become overt only in these neuronal cells.

In conclusion, our study indicates a key role of intracellular PrP misfolding and alterations of AMPAR trafficking in synaptotoxicity and neuronal cell death. It is now important to identify other molecules whose transport is disrupted by the different mutants, and if they contribute to selective neurotoxicity. These important challenges will not only cast light on the physiopathology of genetic prion diseases but also help point the way to devising targeted therapeutic approaches for these devastating disorders.

## Materials and methods

### Ethic statement

Procedures involving animals and their care were conducted in conformity with the institutional guidelines at the Istituto di Ricerche Farmacologiche Mario Negri IRCCS in compliance with national (D.lgs 26/2014; Authorization no. 19/2008-A issued March 6, 2008 by Ministry of Health) and international laws and policies (EEC Council Directive 2010/63/UE; the NIH Guide for the Care and Use of Laboratory Animals, 2011 edition). They were reviewed and approved by the Mario Negri Institute Animal Care and Use Committee, which includes ad hoc members for ethical issues, and by the Italian Ministry of Health (Decreto no. 321/2015-PR and 212/2016-PR). Animal facilities meet international standards and are regularly checked by a certified veterinarian who is responsible for health monitoring, animal welfare supervision, experimental protocols and review of procedures.

### Mice

The production of transgenic mice expressing wild-type, PG14, D177N/V128 and D177N/V128 mouse PrPs has already been reported [11,12,14]. In this study, we used transgenic mice of the Tg(WT-E1^+/+^) line, which express about four times (4X) the endogenous PrP level, Tg(PG14-A3^+/-^) expressing transgenic PrP at ~1X, Tg(CJD-A66^+/-^) and Tg(FFI-26^+/-^) expressing PrP at ~2X. These mice were originally generated on a C57BL/6J X CBA/J hybrid and were then bred with the Zurich I line of *Prnp*^0/0^ mice [58] with a pure C57BL/6J background (European Mouse Mutant Archive, Monterotondo, Rome, Italy; EM:01723). C57BL/6J mice were purchased from Charles River Laboratories.

### Cells

Primary hippocampal neurons were obtained from 2-4-day-old pups, as described [59]. Animals were euthanized, and hippocampi were isolated under a surgical stereomicroscope. Tissues were digested with papain (200 U/ml) in CNDM medium (5.8 mM MgCl_2_, 0.5 mM CaCl_2_, 3.2 mM HEPES, 0.2 mM NaOH, 30 mM K_2_SO_4_ and 90 mM Na_2_SO_4_. pH 7.4, 292 mOsm) supplemented with 0.4% glucose for 30 min at 34°C. Enzymatic activity was blocked with trypsin inhibitors (10 μg/ml, Sigma) in CNDM plus 0.4% glucose for 45 min at room temperature (RT), and the tissue was mechanically dissociated in minimal essential medium α (MEM; Invitrogen) supplemented with 10% fetal bovine serum (FBS) (HyperClone) and 0.4% glucose; 0.5 x 10^5^ cells per cm^2^ were plated on poly-L-lysine-coated (0.1 mg/ml) coverslips in the same medium with penicillin/streptomycin (PenStrep, Lonza, 100U/ml). After attachment, the culture medium was switched to Neurobasal-A (Invitrogen) supplemented with 2% B27 (Invitrogen), 200 mM glutamine and 100U/ml PenStrep. The medium was changed after 7 days in culture.

Primary cerebellar granule neurons were prepared from six-day-old mice. Briefly, cerebella were dissected, sliced into ∼1-mm pieces and incubated in Hank's balanced salt solution (HBSS, Gibco) containing 0.3 mg/ml trypsin (Sigma) at 37°C for 15 min. Trypsin inhibitor (Sigma) was added to a final concentration of 0.5 mg/ml and the tissue was mechanically dissociated by passing through a flame-polished Pasteur pipette. Cells were plated at 350–400,000 cells/cm^2^ on poly-L-lysine (0.1 mg/ml)-coated plates. Cells were maintained in Basal Medium Eagle (Gibco) supplemented with 10% dialyzed FBS (Sigma), penicillin/streptomycin and 25 mM KCl, at 37°C in 5% CO_2_/95% air.

HeLa cells were grown in a 1:1 mixture of Dulbecco’s modified Eagle’s medium (DMEM) and MEM, supplemented with Glutamax (Invitrogen), 10% FBS, non-essential aminoacids (Sigma Aldrich), 100 U/ml penicillin and 100 μg/ml streptomycin (Gibco), and maintained at 37°C in 5% CO_2_/95% air.

### DNA constructs and transfection

pRK expression plasmids encoding the AMPAR subunit myc-GFP-GluR4 (flip) and GluR2 (R607, flop) were provided by Yael Stern-Bach (The Hebrew University of Jerusalem, Israel). A pCDNA3 plasmid encoding GluA2 (R, flop) long C-term was kindly provided by Peter Seeburg (Max-Planck Institute for Neurological Research, Heidelberg, Germany). pCDNA3.1(+) expression plasmids encoding 3F4-tagged mouse PrP, or PrP constructs containing a monomerized version of EGFP inserted after codon 34 of 3F4-tagged mouse PrP have been described [38].

HeLa cells were transfected using the FuGENE HD Transfection Reagent (Promega), according to the manufacturer’s directions and analyzed 48h later. Hippocampal neurons were transfected at days *in vitro* (DIV) 11–13 with Lipofectamine 2000 (Invitrogen) according to the manufacturer’s directions. For dendritic spine analysis neurons were transfected with pEGFP-C1 (Clontech). For analysis of the rectification index upon acute PrP expression cells were transfected with the bigenic pBud-CE4.1 vector (Invitrogen) in which the GFP cDNA had been inserted downstream of the EF-1α promoter, and the cDNA encoding moPrP WT or PG14 downstream of the CMV promoter between the HindIII and XbaI restriction sites [5]. The pBud-CE4.1 vector expressing only GFP was used as control.

### Immunofluorecence

Neurons were fixed in 4% paraformaldehyde (PFA) and 4% sucrose in phosphate buffered saline (PBS) for 8 min, and HeLa cells were fixed with 4% PFA in 200 mM Hepes/NaOH pH 7.4 for 10 min. The following antibodies were used: guinea pig polyclonal anti-Bassoon (Synaptic System 141004, 1:300), rabbit polyclonal anti-shank 2 (Synaptic System 162202, 1:500), rabbit polyclonal anti-βIII-tubulin (Sigma T2200, 1:200), rabbit polyclonal anti-GluA2 (Synaptic System 182103, 1:500), mouse monoclonal anti-GluA2 (Millipore MAB397, 1:200) and mouse monoclonal anti-PrP antibodies 3F4 (1:500) [60] and 98A3 (Wageningen University & Research, 1:500). Secondary antibodies were conjugated with Alexa-488, Alexa-555, or Alexa-633 fluorophores (Invitrogen).

For staining surface AMPARs, live neurons were incubated with a mouse monoclonal antibody directed against the extracellular portion of the receptors (anti-GluA 182411, Synaptic System, 1:100) dissolved in Krebs-Ringers-Henseleit solution (KRH, see below) for 5 min at 37°C and washed three times with fresh KRH. Cells were then fixed and stained as above. Images were acquired using an Olympus FV1000 confocal microscope. For dendritic spines analysis images of proximal dendrites were acquired with a 60X oil immersion objective using a scan format of 1024x1024 pixels. Each image consisted of a stack of images taken through the *z*-plane of the cell. Spines were manually classified with the NeuronStudio software [61], and density was calculated as number of spines/μm.

Synaptic markers were analyzed on single-plane images using ImageJ software (National Institutes of Health, USA). Pixel size was 115 x 115 nm. The minimum puncta size was 3 pixels (0.034 μm^2^). Co-localization of two selected markers was measured using the Boolean function “and” for the selected channels. The resulting image was binarized and used as a co-localization mask to be subtracted from single channels. The numbers of puncta resulting from the co-localization mask subtraction were calculated for each marker. A co-localization fraction was set as the number of co-localizing puncta/total Bassoon puncta.

Surface AMPAR density was analyzed on clusters lying along dendritic branches. Density was calculated as the number of puncta/μm. The fluorescent density of PrP-EGFP and GluA2 on the cell surface of HeLa cells was measured with ImageJ software (National Institutes of Health, USA) and corrected for background. Corrected Integrated Density (IntDen) fluorescence = IntDen–[Area of the region of interest (ROI) X Mean fluorescence of 4 background readings)]. The Pearson’s correlation coefficient and Mander’s co-localization coefficients (M1 and M2) were calculated in selected plasma membrane ROIs using the JACoP plug-in (ImageJ, National Institutes of Health, USA).

For immunofluorescence of mouse cerebellar sections, brains were removed and fixed in ice-cold 4% PFA in PBS for 1h and 30 min, cryoprotected, and frozen at -80°C. Sections (30 μm thick) were cut using a Leica cryostat and incubated at 80°C for 20 min in 10 mM sodium citrate buffer, pH 6.0, for antigen retrieval. After washing in PBS, sections were incubated for 2h in 10% normal goat serum, 1% Triton X-100 in PBS. They were then co-incubated with rabbit polyclonal anti-GluA2 antibody (Synaptic System 182103, 1:200) or rabbit polyclonal anti-GluA4 antibody (Alomone AGC-019, 1:200) and guinea pig polyclonal anti-VGLUT1 antibody (Synaptic System 135304, 1:1000) for 72h at 4°C. After washing with PBS, sections were incubated with biotinylated goat anti-rabbit IgG antibody (Vector, 1:200) and visualized with Alexa Fluor 647 Streptavidin (Molecular Probes Inc., 1:500) and anti-guinea pig Alexa Fluor-conjugated 488-secondary antibody (Molecular Probes Inc., 1:500) in blocking solution for 2h at RT. Immunostained sections were mounted with ProLong Gold antifade reagent (Invitrogen). Images were acquired at 60X with a Zeiss LSM 800 Confocal Laser Scanning Microscope, using the sequential Z-stack acquisition mode and analyzed with NIH ImageJ software. The region of interest (ROI) was drawn manually around the glomerulus based on VGLUT1 signal, and for each glomerulus the areas occupied by GluA2, GluA4 and VGLUT1 signal were measured using the ImageJ software (National Institutes of Health, USA).

### Co-immunoprecipitation

Brain tissue was homogenized in ice-cold co-IP buffer (75 mM NaCl, 1% IGEPAL and protease inhibitor mixture (Sigma), 50 mM Tris-HCl, pH 7.4); 500 μg of protein were diluted in 1 ml of co-IP buffer and passaged five times through a 23-gauge needle. After incubation at 4°C for 30 min on a rotating wheel, the samples were centrifuged at 1000 x g for 3 min to remove non-soluble material. The supernatant was incubated for 1h at room temperature with 20 μl of anti-mouse IgG-conjugated Dynabeads, and then overnight at 4°C with 25 μl/ml of anti-mouse IgG-conjugated Dynabeads coated with the primary antibody anti-PrP 94B4 (Wageningen University & Research) [the primary antibody (1.5 μg) was incubated with 25 μl of anti-mouse IgG-conjugated Dynabeads for 2h at RT in PBS plus 0.1% immunoglobulin-free BSA (Sigma)]. After three washes with 150 mM NaCl, 0.5% IGEPAL, 50 mM Tris-Cl, pH 7.4, immunoprecipitated proteins were eluted in 20 μl DTT-containing Laemmli sample buffer, resolved by standard 7.5% SDS-PAGE and transferred to polyvinylidene fluoride membranes (Immobilon-p, Millipore). Blots were incubated with the antibodies indicated and developed using the ECL system (Illuminata, Millipore). The following antibodies were used: rabbit polyclonal anti-GluA1 (Millipore AB1504, 1:1000), rabbit polyclonal anti-GluA2 (Synaptic System 182103, 1:1000), rabbit polyclonal anti-GluA4 (Alomone AGC-019, 1:200), rabbit polyclonal anti-PrP P45-66 (1:2500) [62]. Peroxidase-conjugated secondary antibodies (Santa Cruz) were used at a dilution of 1:5000.

### Electrophysiology

*Cell culture recordings*. Voltage-clamp whole-cell recordings were obtained from cultured neurons on DIV 13–16 under visual guidance using fluorescence and transmitted light illumination. The extracellular solution (KRH) contained 125 mM NaCl, 5 mM KCl, 1.2 mM MgSO_4_, 1.2 mM KH_2_PO_4_, 25 mM HEPES sodium salt, 2 mM CaCl_2_ and 6 mM glucose. For standard miniature excitatory postsynaptic currents (mEPSCs) recordings, patch pipettes (2.5–4.5 MΩ resistance) made of borosilicate glass were filled with a potassium gluconate-based solution containing 10 mM KCl, 2 mM MgCl_2_, 10 mM HEPES sodium salt, 130 mM potassium gluconate, 1 mM ethylene glycol tetraacetic acid (EGTA), 4 mM Mg-ATP and 0.3 mM Tris-GTP. For rectification index experiments a cesium gluconate-based solution was used, containing 130 mM CsOH, 8 mM CsCl, 2 mM NaCl, 10 mM HEPES sodium salt, 4 mM EGTA, 4 mM Mg-ATP and 0.3 mM Tris-GTP and 120 μM spermine. Synaptic AMPA receptor-mediated currents were measured by holding neurons at a membrane potential of -70 mV with 1 μM TTX (Tocris), 20 μM bicuculline (Tocris) and 20 μM AP5 (Tocris).

Rectification experiments were carried out in extracellular solution containing 10 nM TTX, 20 μM bicuculline and 100 μM AP5. AMPAR-mediated responses were first recorded at -70 mV and then at +50 mV. Cells were recorded at RT in voltage clamp mode using an Axopatch 200b amplifier (Molecular devices) and pClamp-10 software (Axon Instruments). Series resistance ranged from 5 to 20 MΩ and was monitored for consistency during recordings. Cells with leak currents >200 pA, V_m_> -40 mV and mEPSCs frequency < 0.2 Hz were excluded from the analysis. Signals were amplified, sampled at 10 kHz and filtered to 4 KHz, and acquired using the pClamp 10 data acquisition program. Clampfit-10.6 software was used for analysis. Traces were low-pass filtered at 1 KHz. mEPSCs were selected through a template-based analysis and amplitude and frequency were automatically calculated.

*Slice recordings*. To obtain acute cerebellar slices, PG14 and WT mice aged 28–30 days were deeply anesthetized and decapitated. Brains were removed and placed in ice-cold solution. The Krebs solution for slice cutting, recovery and recording contained: 120 mM NaCl, 2 mM KCl, 1.2 mM MgSO4, 26 mM NaHCO3, 1.2 mM KH2PO4, 2 CaCl2, 11 mM glucose. This solution was equilibrated with 95% O_2_-5% CO_2_ (pH 7.4). The cerebellar vermis was isolated, glued to the stage of a vibroslicer (VT1000S Leica), rapidly immersed in ice-cold oxygenated Krebs solution and 200-300-μm-thick slices were cut in the parasagittal plane. The slices were maintained at room temperature before being transferred to a 1.5-ml recording chamber mounted on the stage of a BX51WI upright microscope (Olympus) equipped with a water immersion differential interference contrast objective and an infrared camera (XM10r Olympus).

Granule cells in the internal granular layer and Purkinje cells were voltage clamped with a Multiclamp 700B patch-clamp amplifier (Molecular Devices, Union City, CA) at RT. Patch pipettes were pulled from borosilicate glass capillaries (VWR) and had 8–12 MΩ resistance before seal formation. Pipettes contained: 135 mM CsGluconate, 1 mM EGTA, 10 mM HEPES, 2 mM MgCl2, 4 mM MgATP, and 0.3 mM Tris-GTP, (pH 7.4). Rectification experiments were carried out in extracellular solution containing 10 nM TTX, 20 μM bicuculline and 100 μM AP5. AMPAR-mediated responses were first recorded at -70 mV and then at +50 mV. Synaptic currents were low-pass filtered at 2 kHz, sampled at 10 kHz and analyzed with pClamp/Digidata 1440A (Molecular Devices). Clampfit-10.6 software was used for analysis.

### Calcium imaging

Hippocampal neurons on 13–16 DIV were loaded with 10 mM Oregon Green 488 BAPTA-1 AM (Molecular Probes) in culture medium for 45 min at 37°C, washed in KRH and transferred to the recording chamber of an IX-71 inverted microscope (Olympus, Hamburg, Germany) equipped with an EMCCD (electron-multiplying CCD) camera (Quantem 512 × 512, Photometrics). A light-emitting diode single LED (Cairn Research Optoled; 470 nm) and a related GFP filter were used for illumination; 16-bit images were captured with a 20X objective (NA: 0.8). ROIs of about 15-pixel diameter (corresponding to ~12 μm) were drawn on the cell cytoplasm of virtually all the cells in the recorded field. Time-lapse recording of calcium dynamics was done at an acquisition rate of 5 Hz for 600 sec and analyzed off-line with the MetaFluor software (Molecular Devices). Cerebellar granule neurons were loaded with 5 μM Fura-2 pentacetoxymethylester (Life Technologies) and recorded with an inverted microscope (Axiovert 100; Zeiss, Oberkochen, Germany) equipped with a Polychrome IV (TILL Photonics, Germany). After excitation at 340 and 380 nm, the emitted light was acquired in selected ROI corresponding to neuronal somata at 505 nm at 1–2 Hz. The exposure time was 10 msec for 340 nm and 20 msec for 380 nm. A constant value corresponding to the emitted light at 340 nm excitation in the empty area of the coverslip was subtracted as background. After a period for baseline acquisition, neurons were stimulated with 30 μM AMPA (Tocris) for 30 sec with 1 μM TTX, 100 μM Cd^2+^, 100 μM AP5 and 20 μM nifedipine (Tocris). Calcium responses were measured as Δ*F*(*F*_max_− *F*_0_) in relation to the baseline (*F*_0_). A total of 10–15 neurons were analyzed for each field.

### Organotypic cerebellar slices

Cerebella were dissected from 9-10-day-old mice and transferred to a container with liquid agarose [2% low-melting point agarose in Geys Balanced Salt Solution with 33.33 mM glucose and 1 mM kynurenic acid (GBSSK)]. After cooling on ice, the agarose blocks were glued onto the vibratome disc, which was then placed in ice-cold GBSSK in the buffer reservoir of the vibratome (VT 1000S Leica). Several 350 μm sagittal slices were cut at low frequency (setting 6–7) and speed (setting 5–6). Slices were collected in a Petri dish containing ice-cold GBSSK and released from the agarose under a stereomicroscope using fine forceps. All subsequent steps were carried out under sterile conditions in a cell culture hood. Three to four slices were placed in a Millicell Cell Culture Insert (Millipore) in a six-well cell culture plate. Wells contained 1 ml of slice culture medium consisting of 50% MEM (Invitrogen), 22.5% HBSS, 25% horse serum (heat-inactivated; Gibco/Life Technologies), 50 U/ml penicillin, 50 μg/ml streptomycin (Invitrogen), 0.6% glucose, and 2 mM GlutaMax (Invitrogen). Slice cultures were kept under standard cell culture conditions (37°C, 5% CO_2_/95% air) in a humidified atmosphere. The culture medium was changed every other day.

Slice cultures were exposed to 50 μM AMPA on DIV 15 with or without 25 μM CNQX. After 24h slices were incubated with 2 μM PI in culture medium for 30 min in a cell culture incubator. Slices were examined with an Olympus BX-61 Virtual Stage microscope, interfaced with VS-ASW-FL software (Olympus), using a 4X objective. Positive nuclei were counted with the same settings for brightness and diameter for each image, using ImageJ software (National Institutes of Health, USA).

### Immunoelectron microscopy

Immunoelectron microscopy of PrP in cultured cerebellar granule neurons, quantification of gold particles in the different compartments of the secretory pathway, and analysis of total cell, ER and Golgi volumes, were all done as described [14].

### Statistical analysis

Results were statistically analyzed using Excel (Microsoft) or Prism 7 (GraphPad, Software Inc.). After testing data for normal distribution with the Kolmogorov-Smirnov test, the appropriate statistical tests were used (see Fig legends). Data are presented as mean ± standard error of the mean (SEM) of the number of elements analyzed. P < 0.05 was considered statistically significant.

## Supporting information

S1 Data Excel spreadsheet containing, in separate sheets, the underlying numerical data for Figure panels 1C, 1D, 1F, 1G, 1I, 1J, 1K, 2C, 2D, 3A, 3B, 3C, 3D, 3E, 3F, 3G, 3I, 3J, 4B, 4C, 5B, 5D, 5F, 5G, 5I, 5K, 6A, 6C, 6D, 6E, 6G, 7A, 7B, 7C and 8B. (XLSX)

S1 Fig Colocalization of PrP and GluA2 on the plasma membrane of transfected HeLa cells. (A) Representative confocal images of HeLa cells co-transfected with plasmids encoding WT PrP-EGFP and GluA2 showing PrP (green) and GluA2 (red) fluorescence on the plasma membrane. The dotted white square region is magnified and shown in the inset. Scale bar 10 μm. (B) Summary of Pearson’s correlation coefficient and Mander’s coefficient values (M1 and M2). M1: PrP fraction that co-localizes with GluA2; M2: GluA2 fraction that co-localizes with PrP. Data are the mean ± SEM of 28 cells from six independent experiments. Pearson’s, 0.80±0.02; M1, 0.53±0.03; M2, 0.58±0.03.
(TIF)

S2 Fig IEM-1460 reduces AMPA-induced mortality in CJD neurons. Hippocampal cultures from WT (A), FFI (B) and CJD (C) mice were treated with 300 μM AMPA or 300 μM AMPA and 50 μM IEM-1460. After 24h cells were incubated with Hoechst 33258 (10 μg/ml) and propidium iodide (PI; 2 μg/ml) for 30 min and mortality was calculated as PI/Hoechst 33258 positive nuclei. Data are the mean ± SEM of 8–12 replicates from three to four independent experiments. WT AMPA, 1.00±0.09; WT AMPA-IEM, 1.06±0.13; FFI AMPA, 1.00±0.11; FFI AMPA-IEM, 0.89±0.15; CJD AMPA, 1.00±0.06; CJD AMPA-IEM, 0.68±0.10. *p < 0.05, two-tailed unpaired t-test.
(TIF)

S3 Fig PG14 PrP accumulates in the endoplasmic reticulum of cerebellar granule neurons. Cultures of cerebellar granule neurons from Tg(WT) and Tg(PG14) mice were fixed and labeled with anti-PrP monoclonal antibody 12B2 using the gold-enhance protocol. (A) WT PrP is mostly found at the plasma membrane (arrows); some staining is also seen in endosomes (arrowheads). (B) PG14 PrP is mostly in the ER (arrows), whose cisternae appear enlarged and electron-dense. Scale bar 250 nm. (C) Quantification of gold particles in different cell compartments. PM, plasma membrane. Data are the mean ± SD of at least 10 cells per specimen. WT (ER, 2.33±0.26; Golgi, 2.87±0.27; PM, 86.20±1.85; Endosomes, 8.58±1.83); PG14 (ER, 62.46±7.54; Golgi, 14.94±8.88; PM, 20.56±4.17; Endosomes, 2.01±0.35). (D) Quantification of ER and Golgi volumes of cultured cerebellar granule neurons. Data are the mean ± SD of at least 10 cells per specimen. WT (ER, 5.73±1.90; Golgi, 1.53±0.40); PG14 (ER, 13.52±2.21; Golgi, 2.82±1. 75). Data for Tg(WT) neurons in C and D are from [14].
(TIF)

S4 Fig Cerebellar granule neurons express basal levels of GluA2-lacking, calcium permeable AMPA receptors. (A) Analysis of calcium peaks and (B) representative traces. Cerebellar granule neurons form WT mice cultured for 8 days in high-K^+^ medium, were loaded with the calcium-sensitive dye Fura-2, then recorded by single cell calcium imaging in the presence of 1 μM TTX, 100 μM Cd^2+^, 100 μM AP5 and 20 μM nifedipine after exposure to 30 μM AMPA for 30 seconds. After AMPA wash-out 50 μM IEM-1460 was added, neurons allowed to recover for five minutes and stimulated with AMPA again. Data are the mean ± SEM of 25 cells from three fields. AMPA, 0.39±0.05; AMPA+IEM, 0.12±0.02; ****p < 0.0001 by two-tailed, Wilcoxon matched-pairs signed rank test.
(TIF)

S5 Fig AMPA induces apoptosis in cerebellar granule neurons. (A) Cultures of cerebellar granule neurons from C57BL/6J mice were exposed to 300 μM AMPA for 24h. Cells were fixed and subjected to TUNEL staining (DeadEnd Fluorometric TUNEL System, Promega), then reacted with Hoechst 33258 to stain cell nuclei. Scale bar 100 μm. (B) TUNEL-positive cells were counted and expressed as percentages of the total cells identified with Hoechst 33258. CT, 0.67%; AMPA, 7.99%.
(TIF)
