# Correction: Mutant prion proteins increase calcium permeability of AMPA receptors, exacerbating excitotoxicity

**DOI:** 10.1371/journal.ppat.1009174

**Published:** 2020-12-22

**Authors:** Elsa Ghirardini, Elena Restelli, Raffaella Morini, Ilaria Bertani, Davide Ortolan, Fabio Perrucci, Davide Pozzi, Michela Matteoli, Roberto Chiesa

There is an error in affiliation 1 for authors Elsa Ghirardini, Raffaella Morini, Fabio Perrucci, Davide Pozzi and Michela Matteoli. The correct affiliation 1 is: Laboratory of Pharmacology and Brain Pathology, Humanitas Clinical and Research Center – IRCCS, Rozzano— Milan, Italy.
