# Supplementary figures and images for: Mutant prion proteins increase calcium permeability of AMPA receptors, exacerbating excitotoxicity
Source: PLoS Pathog. 2020 Jul 16;16(7):e1008654. doi: 10.1371/journal.ppat.1008654 (PMC7365390; doi:10.1371/journal.ppat.1008654)

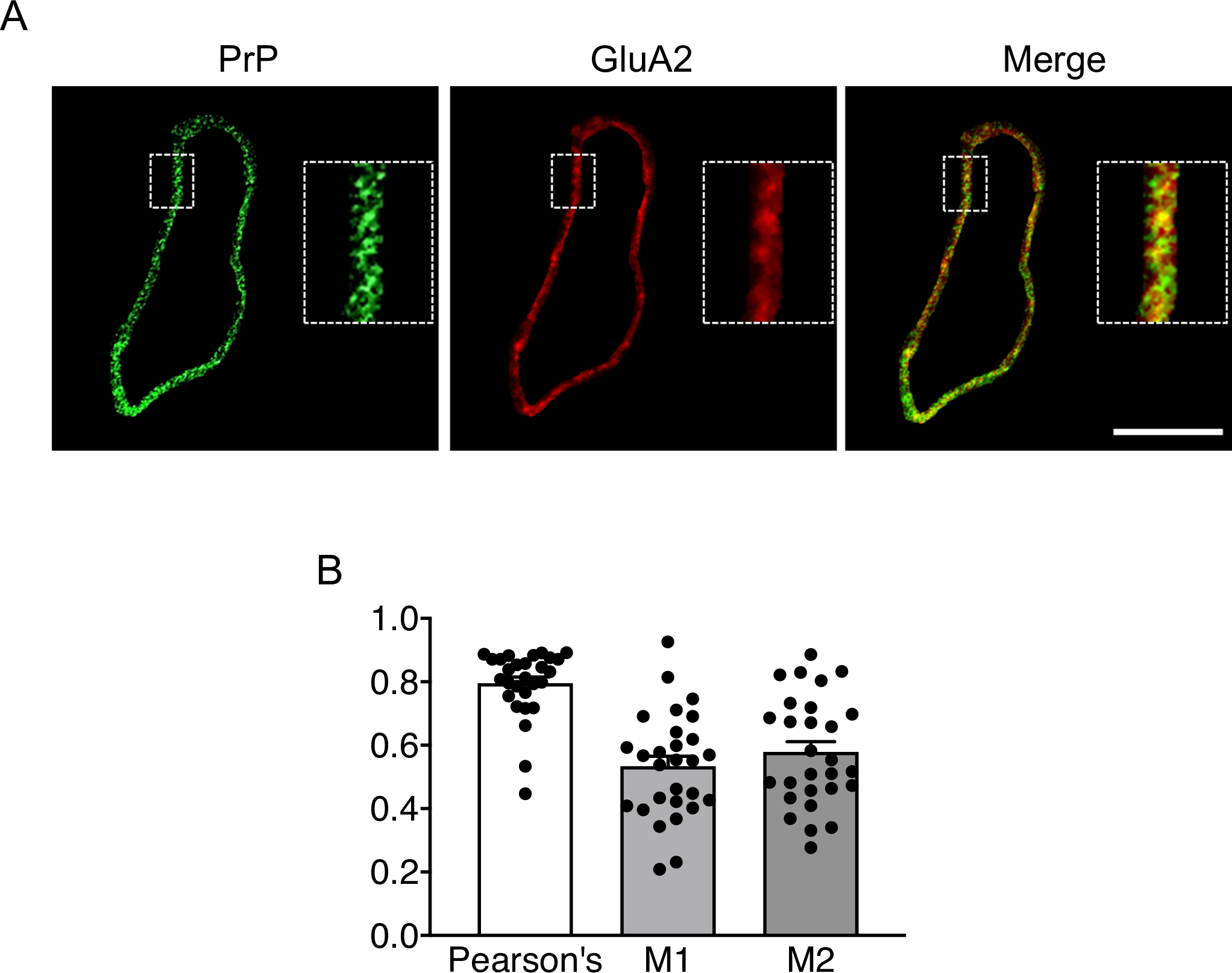

Supplement: S1 Fig — (A) Representative confocal images of HeLa cells co-transfected with plasmids encoding WT PrP-EGFP and GluA2 showing PrP (green) and GluA2 (red) fluorescence on the plasma membrane. The dotted white square region is magnified and shown in the inset. Scale bar 10 μm. (B) Summary of Pearson’s correlation coefficient and Mander’s coefficient values (M1 and M2). M1: PrP fraction that co-localizes with GluA2; M2: GluA2 fraction that co-localizes with PrP. Data are the mean ± SEM of 28 cells from six independent experiments. Pearson’s, 0.80±0.02; M1, 0.53±0.03; M2, 0.58±0.03. (TIF) [file ppat.1008654.s002.tif]

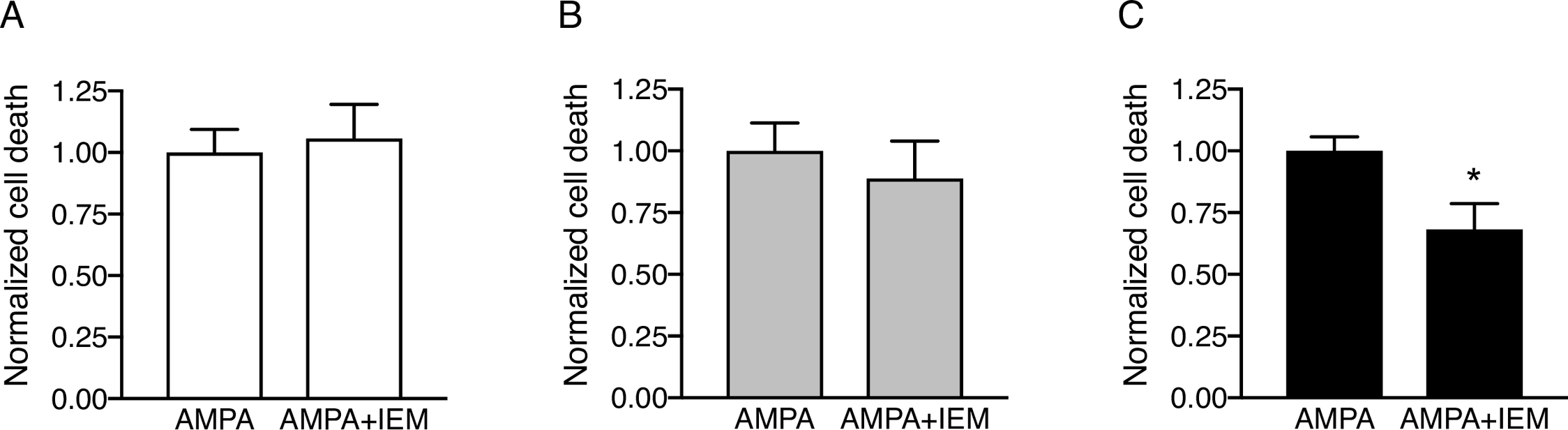

Supplement: S2 Fig — Hippocampal cultures from WT (A), FFI (B) and CJD (C) mice were treated with 300 μM AMPA or 300 μM AMPA and 50 μM IEM-1460. After 24h cells were incubated with Hoechst 33258 (10 μg/ml) and propidium iodide (PI; 2 μg/ml) for 30 min and mortality was calculated as PI/Hoechst 33258 positive nuclei. Data are the mean ± SEM of 8–12 replicates from three to four independent experiments. WT AMPA, 1.00±0.09; WT AMPA-IEM, 1.06±0.13; FFI AMPA, 1.00±0.11; FFI AMPA-IEM, 0.89±0.15; CJD AMPA, 1.00±0.06; CJD AMPA-IEM, 0.68±0.10. *p < 0.05, two-tailed unpaired t-test. (TIF) [file ppat.1008654.s003.tif]

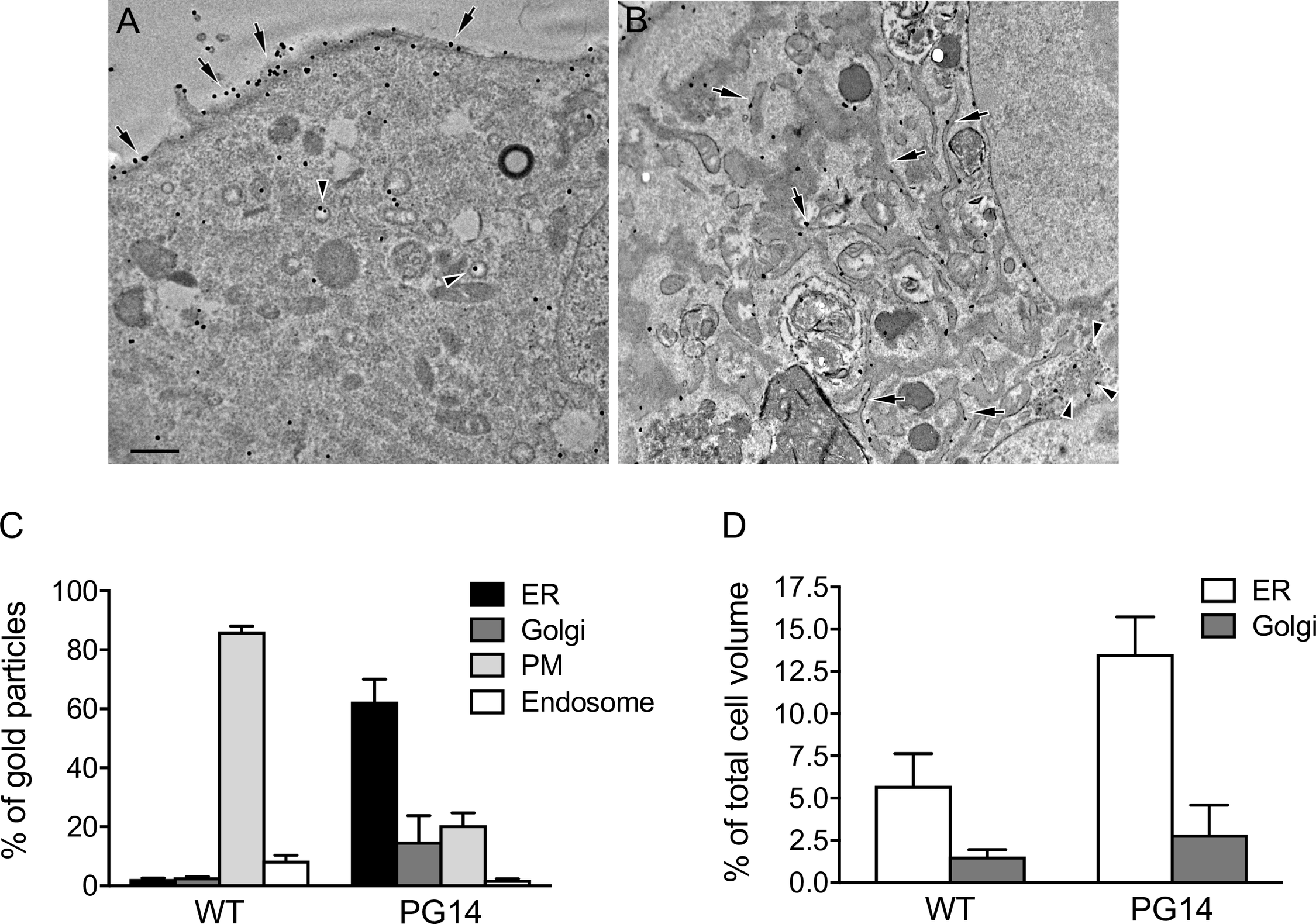

Supplement: S3 Fig — Cultures of cerebellar granule neurons from Tg(WT) and Tg(PG14) mice were fixed and labeled with anti-PrP monoclonal antibody 12B2 using the gold-enhance protocol. (A) WT PrP is mostly found at the plasma membrane (arrows); some staining is also seen in endosomes (arrowheads). (B) PG14 PrP is mostly in the ER (arrows), whose cisternae appear enlarged and electron-dense. Scale bar 250 nm. (C) Quantification of gold particles in different cell compartments. PM, plasma membrane. Data are the mean ± SD of at least 10 cells per specimen. WT (ER, 2.33±0.26; Golgi, 2.87±0.27; PM, 86.20±1.85; Endosomes, 8.58±1.83); PG14 (ER, 62.46±7.54; Golgi, 14.94±8.88; PM, 20.56±4.17; Endosomes, 2.01±0.35). (D) Quantification of ER and Golgi volumes of cultured cerebellar granule neurons. Data are the mean ± SD of at least 10 cells per specimen. WT (ER, 5.73±1.90; Golgi, 1.53±0.40); PG14 (ER, 13.52±2.21; Golgi, 2.82±1. 75). Data for Tg(WT) neurons in C and D are from [14]. (TIF) [file ppat.1008654.s004.tif]

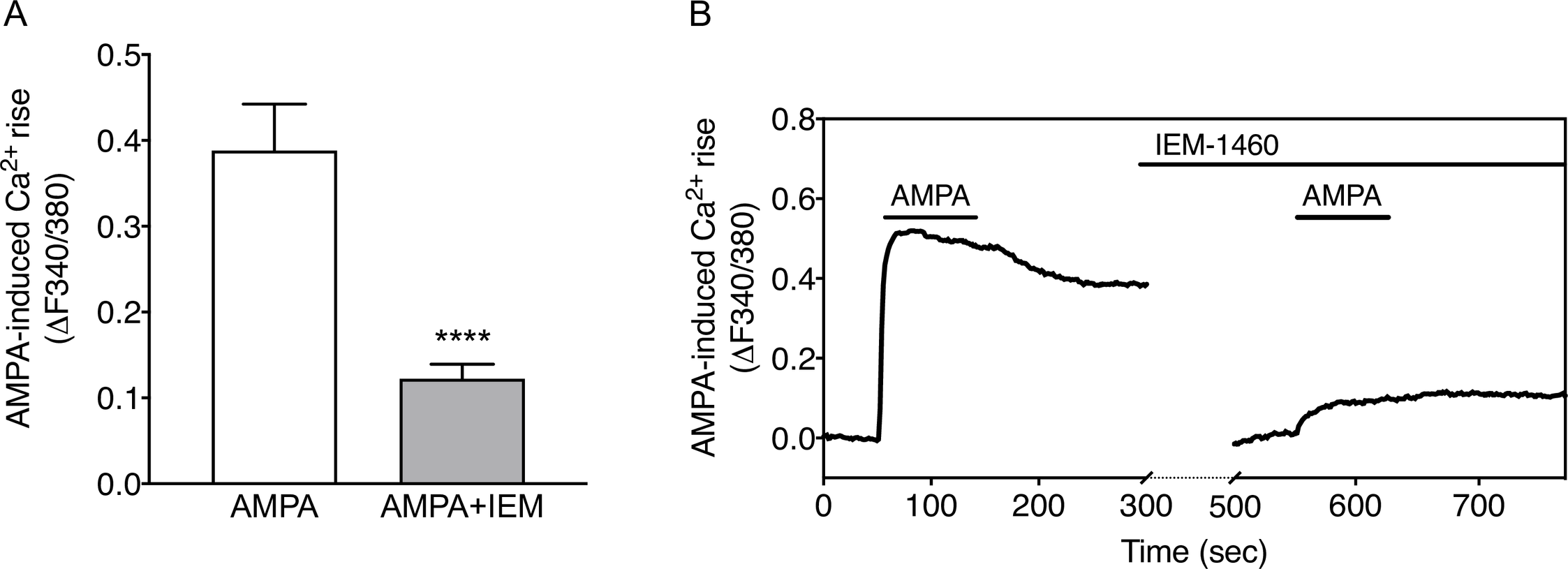

Supplement: S4 Fig — (A) Analysis of calcium peaks and (B) representative traces. Cerebellar granule neurons form WT mice cultured for 8 days in high-K+ medium, were loaded with the calcium-sensitive dye Fura-2, then recorded by single cell calcium imaging in the presence of 1 μM TTX, 100 μM Cd2+, 100 μM AP5 and 20 μM nifedipine after exposure to 30 μM AMPA for 30 seconds. After AMPA wash-out 50 μM IEM-1460 was added, neurons allowed to recover for five minutes and stimulated with AMPA again. Data are the mean ± SEM of 25 cells from three fields. AMPA, 0.39±0.05; AMPA+IEM, 0.12±0.02; ****p < 0.0001 by two-tailed, Wilcoxon matched-pairs signed rank test. (TIF) [file ppat.1008654.s005.tif]

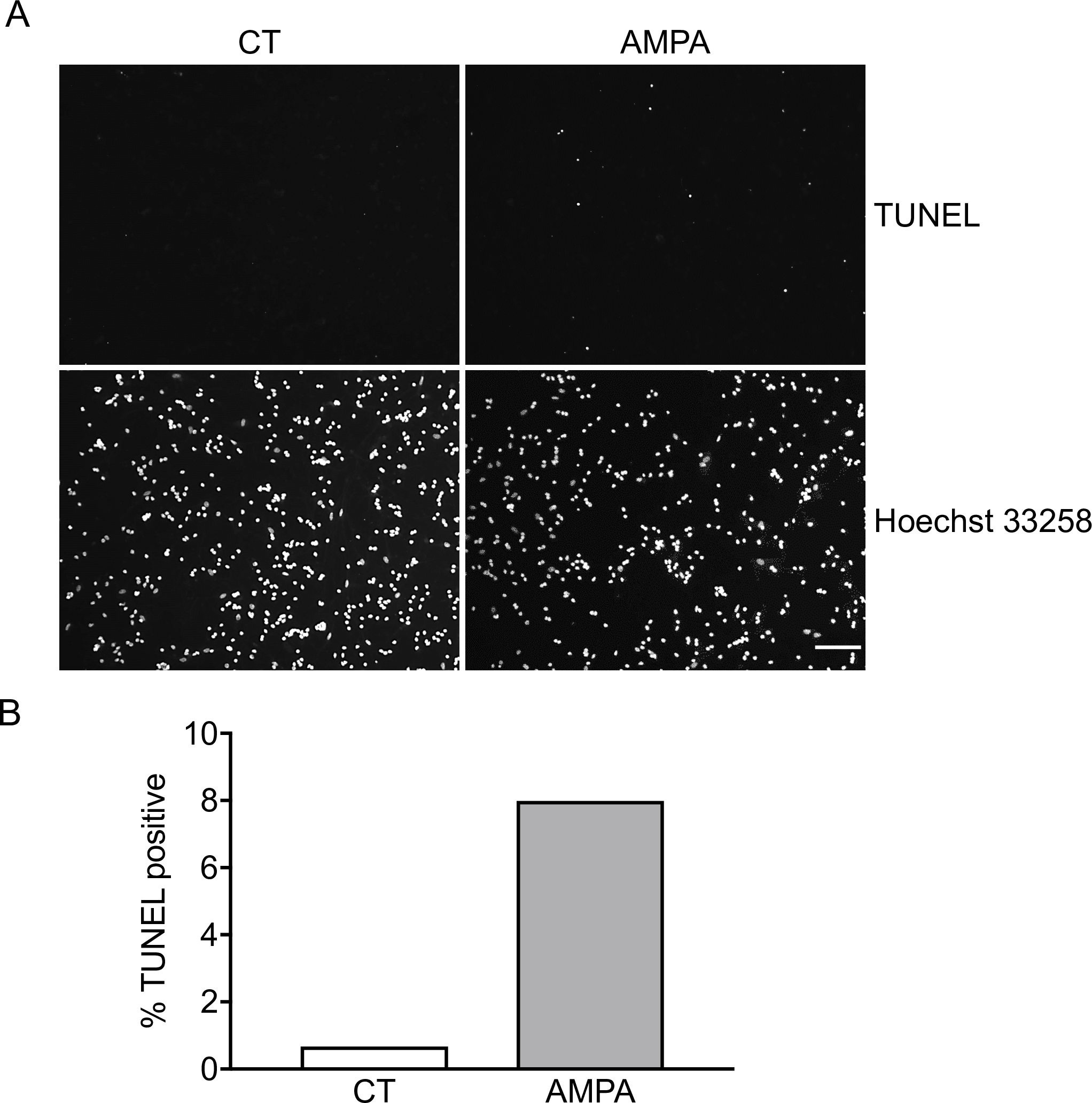

Supplement: S5 Fig — (A) Cultures of cerebellar granule neurons from C57BL/6J mice were exposed to 300 μM AMPA for 24h. Cells were fixed and subjected to TUNEL staining (DeadEnd Fluorometric TUNEL System, Promega), then reacted with Hoechst 33258 to stain cell nuclei. Scale bar 100 μm. (B) TUNEL-positive cells were counted and expressed as percentages of the total cells identified with Hoechst 33258. CT, 0.67%; AMPA, 7.99%. (TIF) [file ppat.1008654.s006.tif]
